# Predicting Peptide Structures in Native Proteins from Physical Simulations of Fragments

**DOI:** 10.1371/journal.pcbi.1000281

**Published:** 2009-02-06

**Authors:** Vincent A. Voelz, M. Scott Shell, Ken A. Dill

**Affiliations:** 1 Department of Chemistry, Stanford University, Stanford, California, United States of America; 2 Department of Chemical Engineering, University of California Santa Barbara, Santa Barbara, California, United States of America; 3 Department of Pharmaceutical Chemistry, University of California San Francisco, San Francisco, California, United States of America; Rensselaer Polytechnic Institute, United States of America

## Abstract

It has long been proposed that much of the information encoding how a protein folds is contained locally in the peptide chain. Here we present a large-scale simulation study designed to examine the extent to which conformations of peptide fragments in water predict native conformations in proteins. We perform replica exchange molecular dynamics (REMD) simulations of 872 8-mer, 12-mer, and 16-mer peptide fragments from 13 proteins using the AMBER 96 force field and the OBC implicit solvent model. To analyze the simulations, we compute various contact-based metrics, such as contact probability, and then apply Bayesian classifier methods to infer which metastable contacts are likely to be native vs. non-native. We find that a simple measure, the observed contact probability, is largely more predictive of a peptide's native structure in the protein than combinations of metrics or multi-body components. Our best classification model is a logistic regression model that can achieve up to 63% correct classifications for 8-mers, 71% for 12-mers, and 76% for 16-mers. We validate these results on fragments of a protein outside our training set. We conclude that local structure provides information to solve some but not all of the conformational search problem. These results help improve our understanding of folding mechanisms, and have implications for improving physics-based conformational sampling and structure prediction using all-atom molecular simulations.

## Author Summary

Proteins must fold to unique native structures in order to perform their functions. To do this, proteins must solve a complicated conformational search problem, the details of which remain difficult to study experimentally. Predicting folding pathways and the mechanisms by which proteins fold is thus central to understanding how proteins work. One longstanding question is the extent to which proteins solve the search problem locally, by folding into sub-structures that are dictated primarily by local sequence. Here, we address this question by conducting a large-scale molecular dynamics simulation study of protein fragments in water. The simulation data was then used to optimize a statistical model that predicted native and non-native contacts. The performance of the resulting model suggests that local structuring provides some but not all of the information to solve the folding problem, and that molecular dynamics simulation of fragments can be useful for protein structure prediction and design.

## Introduction

It has long been proposed that much of the information encoding how a protein folds is contained locally in the peptide chain. Indeed, the success of fragment insertion methods for *ab initio* folding algorithms often relies on the predicted structures of small peptide pieces of the target protein [1],[2]. To what extent do the conformations of peptide fragments in water predict native conformations in proteins? We are interested in this question for at least two reasons. First, accurate local structure predictions from all-atom simulations of small peptide fragments of proteins in water may be useful for physics-based “divide and conquer” strategies for protein structure prediction, such as in the “zipping and assembly” method [3]–[5]. Physics-based methods for prediction offer several potential advantages over database-driven methods, such as the ability to simulate dynamics and predict folding pathways, using transferrable forcefield models which can be applied to a wide range of other problems. Second, this work informs an ongoing discussion about how much of the native structure of a protein is encoded within local sequence information alone [6],[7]. In the “framework mechanism” [8], for example, local information is sufficient to reduce the conformational searching enormously. On the other hand, protein folding is highly cooperative, so models such as the “nucleation-condensation model” indicate that secondary and tertiary structure may form concurrently [9]. Elucidating the role of local structure can help improve our understanding of protein folding mechanisms in general.

The question we raise here is not about the success rates of secondary structure predictions. Secondary structure prediction methods such as PSIPRED use knowledge bases of known native structures and can achieve prediction success rates near 80% (as judged by 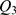 scores) [10]. Here we ask a question of physics. If you knew the physical structure of a peptide in water, rather than in a database of native protein structures, would it predict the conformation of the same peptide in the protein's native structure? As an approximation to the physics, we rely on all-atom force field simulations here. Much work has shown that simulations using current all-atom forcefields can sufficiently and accurately reflect the underlying physics [11]–[13].

There are previous studies using molecular dynamics simulations of peptide fragments for structure prediction. Bystroff and Garde performed 10-ns explicit-water simulations using the AMBER ff94 forcefield for 64 8-residue fragments to show that observed helicity correlates well with I-sites predictions [14]. Ho and Dill performed REMD simulations using the AMBER ff96 forcefield with the GB model of Tsui and Case for 133 8-residue fragments from six different proteins to identify regions of local native-like structure that could serve as folding nuclei [15]. Here, we perform much more extensive tests, over a larger data set and with multiple metrics, made possible by using a high-efficiency search method, called ZAM (Zipping and Assembly Method), that samples the important parts of conformational space. We perform 872 independent simulations of 8-mer, 12-mer, and 16-mer fragments from 13 test proteins, for a total of 8.7 CPU-years of simulation time, which is, as far as we know, the largest set of fragment simulations performed to date. We use the AMBER ff96 forcefield [16] with the GB implicit solvent model mode of Onufriev, Bashford and Case [17], which we have found to predict the structures of a set small peptides with better accuracy than other combinations of AMBER forcefields with GB solvation models [13]. This forcefield has been used with the ZAM conformational search algorithm to predict protein structures in the CASP7 competition [4].

## Results

### The Fragment Simulations Sample Around Native-Like Structures

To what extent did our simulations of peptide fragments sample native-like structures? From the native structures of our target sequences, we determined alpha helical and tight turn types across each target sequence using the secondary structure classification algorithm STRIDE [18]. The turn types were further divided into two groups, one for turns in beta-hairpins, and one for everything else. We filtered the dataset for fragments that were known to have at least 7, 12, and 16 native contacts respectively for 8-, 12- and 16-mers. This selects a subset of fragments with known native secondary structures to which we could compare our simulation data.

**Figure 1:**
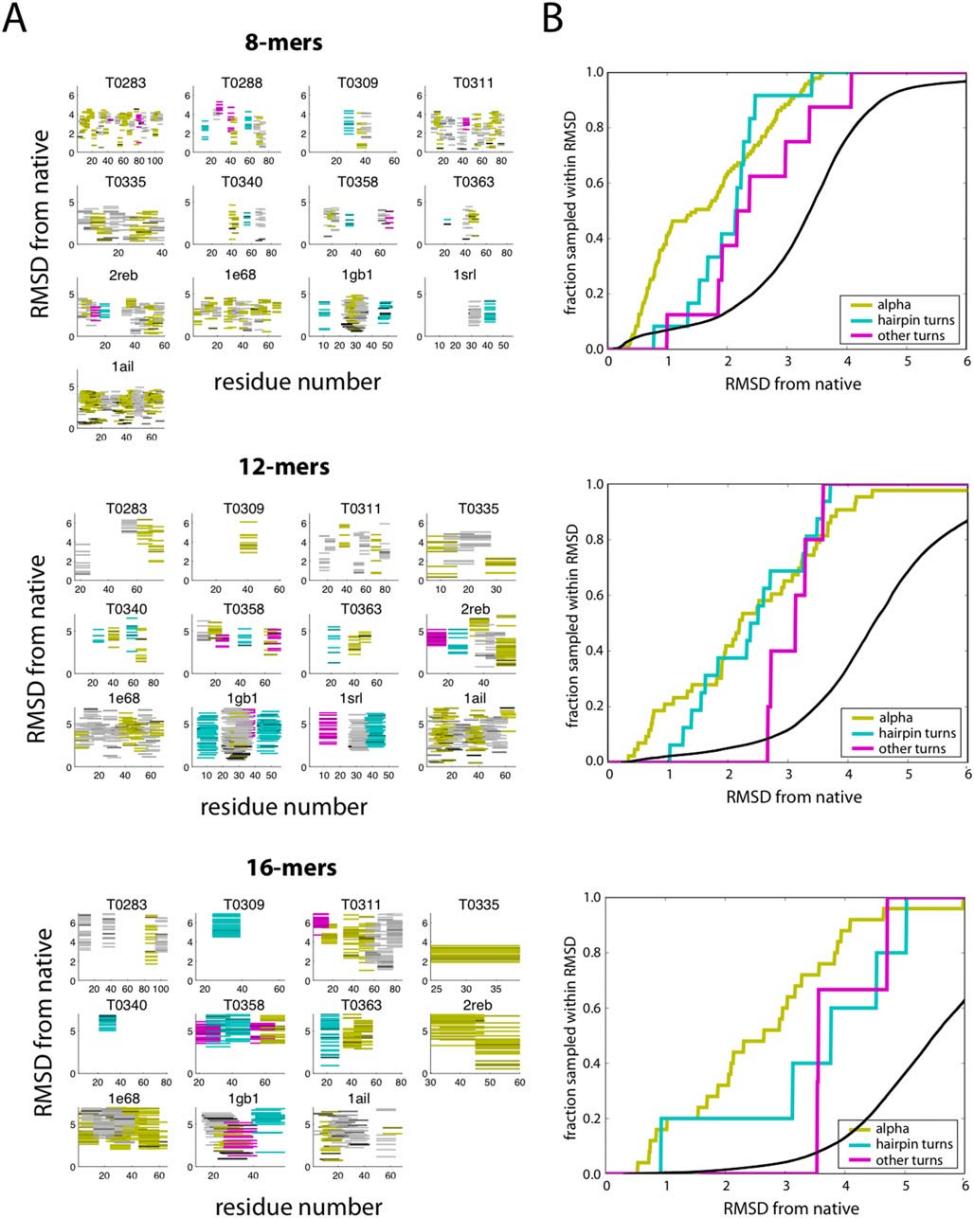
Cluster conformations from fragment simulations sample native-like states. (A) For each target sequence and fragment length, the C-alpha RMSD-to-native values (in Å) for all representative cluster conformations along the target sequence are shown. Each line on the plot corresponds to a cluster conformation, color-coded by native secondary structure: alpha-helix (yellow), beta-hairpin (cyan), or other turn types (magenta). The relative shading of the lines are proportional to the population fraction. The horizontal axis is the sequence position along the protein chain. (B) The fraction of cluster conformations that sample within a particular RMSD-to-native, across all fragment simulations of a given chain length. For comparison, the black line shows the results for a random distribution of C-alpha RMSD values calculated from native protein structures (see Methods).

We find that the fragment simulations sample diverse structures. Conformational clustering (see Methods) produces about 10 representative cluster conformations for each fragment simulation. Figure 1A shows, for each target sequence and fragment length, the C-alpha RMSD-to-native values for all representative cluster conformations along the target sequence.

These fragments typically sample native-like conformations. Figure 1B plots the fraction of cluster conformations that sample within a given RMSD of the native conformation. It is not clear that native-like sampling would necessarily be expected; it depends on the relative importance of the tertiary context [7],[19]. Nevertheless, we find that about 65% of 8-mer alpha helical conformations are within 2.0Å RMSD of the native state, and about 40% of 12-mers and 16-mers are within this range. For comparison, a random distribution of C-alpha RMSD calculated from native protein structures contains only about 10% of 8-mers, 5% of 12-mers, and 2% of 16-mer conformations with RMSD within 2.0Å RMSD (see Methods). About 40% of 8-mer and 12-mer beta hairpin turns were within 2.0Å of the native structure, and 40% of 16-mer hairpins were within 3.0Å of the native structure (only about 5% of random native 16-mer conformations are within 3.0Å RMSD). These results suggest that beta hairpins are more context-dependent, while helices are more generally defined locally. Also, we observe that beta hairpins show more structural variation in general than helices, due to the nonlocal contact topology.

Does running longer simulations lead to more native-like structures? We found this not to be the case. On seven different hairpin fragments, we performed 20 REMD simulations (with and without various contact constraints) for a total of 100 ns (Text S1). In these tests, we simulated both hairpins that corresponded to native structures, and “decoy” hairpins that were predicted by our simulations, but did not correspond to native structures. We conclude that longer simulation does not produce more native-like structures in our simulations. This could be for several reasons: (1) simulations longer than 100 ns would be needed, or (2) the physical model we used is not perfect [13],[20]), or (3) because tertiary context is needed to drive them into their native states. While this work does not attempt to fully resolve these issues, it does establish a lower bound on the extent to which simulations of peptide fragments predict native-like structures, which we find here to be considerable.

### Optimal Classification Models and Contact Metrics

Our data provides an opportunity to draw inferences about what physical properties of intrachain contacts are predictive of whether a peptide conformation is native or not. To do this, we train probabilistic classifier models on several contact metrics, and interrogate the results. For each set of simulated fragments (8-mers, 12-mers, and 16-mers), we explored two kinds of per-contact classification models: a naive Bayes model and a logistic regression model (see Methods). To find the most predictive classifier, each model was trained on all possible combinations of per-contact metrics (defined in Methods) calculated from the simulations.

Which classification model best predicts native or non-native contacts from short fragment simulations? In all cases, the logistic regression model gave better classifications than the corresponding naive Bayes model, thus we present only the results from the logistic regression models. Also in all cases, contacts defined by a 7Å distance cutoff performed significantly worse than an 8Å cutoff, thus we only present results from the latter case. The best logistic regression coefficients for 8-mers, 12-mers, and 16-mers are shown in Table 1.

**Table 1:** The coefficients for the best logistic regression models.

Length	Distance Method	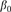 Prior	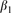 CPROB	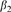 DPROF	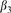 MSTAB	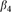 MCOOP	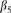 MESO
8	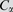	−2.4388±0.1004	2.6401±0.2354	—	−0.0524±0.019	—	—
12	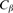	−2.311±0.057	2.594±0.157	—	−0.0363±0.0074	−0.0327±0.0085	—
16	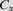	−2.166±0.033	2.194±0.113	0.093±0.0064	−0.025±0.0037	0.0079±0.0041	—

**Figure 2:**
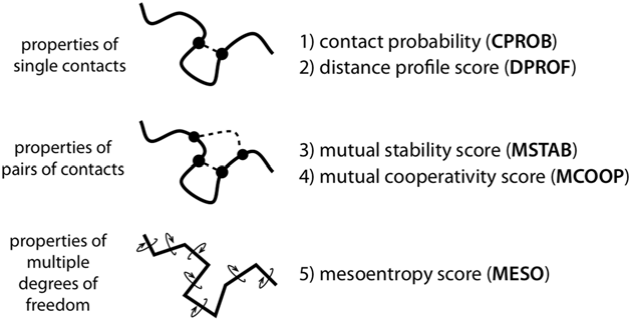
A summary of the contact metrics examined in this study. Each metric is calculated on a per-contact basis from the simulation data. Further details are in Methods.

**Figure 3:**
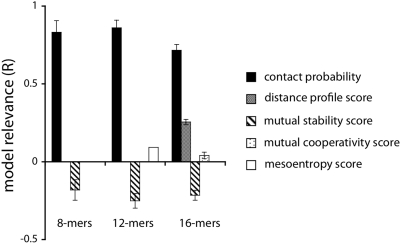
The model relevances 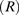 for each contact metric in the best 8-mer, 12-mer, and 16-mer linear regression models. The 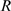 values show that contact probability (CPROB) is the most important metric in predicting whether a contact observed in the computer simulations is likely to be in the native structure of the protein. The model relevance 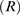 of a contact metric 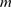 is defined as 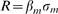, where 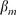 is the logistic regression coefficient for the metric, and 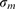 is the standard deviation of the metric.

What metrics are the best predictors of whether a simulated fragment has formed native contacts? We examined several metrics (see Methods), each calculated on a per-contact basis from the simulation data (Figure 2): (1) contact probability (CPROB), the equilibrium probability of a given contact, (2) a distance profile score (DPROF) quantifying interresidue probabilities as a function of distance, (3) a mutual stability score (MSTAB) quantifying the joint probability of a contact when making pairs with other contacts, (4) a mutual cooperativity score (MCOOP) quantifying cooperative interactions made with other contacts, and (5) a mesoentropy score (MESO), which is a measure of the backbone conformational entropy. Since the numerical values of the five contact metrics can differ by orders of magnitude, we obtain a better sense of the relative importance of the different contact metrics by computing the model relevance 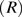, which we define as 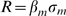, where 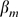 is the logistic regression coefficient for contact metric 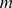, and 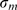 is the standard deviation of the metric. The 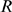 values calculated for each regression coefficient show that the most predictive metric is the contact probability (Figure 3).

This is interesting because it might be expected that including multi-body terms would be more predictive than just the pairwise contact formation probability, since protein stability is likely to involve non-additivities that could only be captured in complex terms. Instead, we find that simple pairwise terms are the most predictive, with the multi-body terms producing small negative regression coefficients. The negative coefficients can be interpreted as providing a slight correction to the over-counting due to correlation between pairwise contact probability terms.

**Figure 4:**
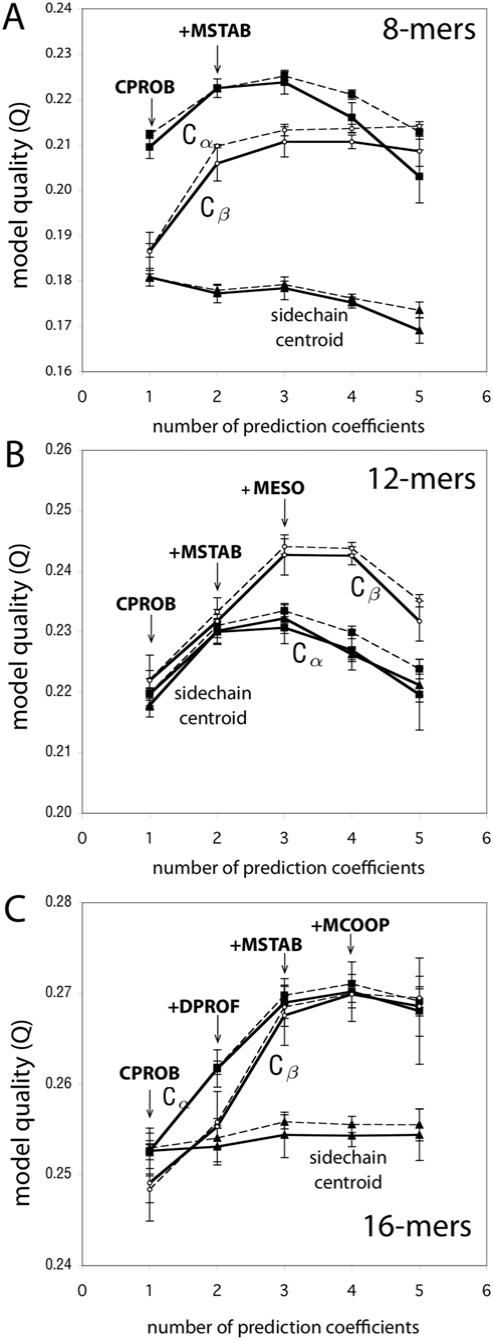
Testing and training curves for the logistic regression models. Results are shown for models built from the (A) 8-mer simulation data, (B) 12-mer data, and (C) 16-mer data. For each contact definition we tested (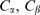, and sidechain-centroid), shown is the model quality (Q) for a series of models, calculated from the training data (dotted) and the testing data (solid) (see Methods for details). The larger the 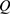 value, the more predictive the model. From left to right, the model quality (Q) for the best 1-, 2-, 3-, 4-, and 5-metric regression models are plotted, labeled with the sequence of additional metrics that increasingly improve the model quality.

Figure 4 shows the results of increasing the number of prediction coefficients. These curves make essentially three points. First, the best first approximation, i.e., the most predictive single term, as noted above, is CPROB, the contact probability. Second, the figure shows that the predictive power of the model increases by adding up to two additional terms. However, the added value in predictive power is quite small. And, third, it shows that adding further terms to the model, beyond three, worsens the predictive power.

We also tested whether we could obtain better classification models by training on local contacts (or nonlocal contacts) alone. We found that, overall, the classification success for the local-only or nonlocal-only data was comparable, but never as high as the classification success using the combined data (see Text S1).

### Predicting Native Contacts and Conformations from Fragment Simulations

**Figure 5:**
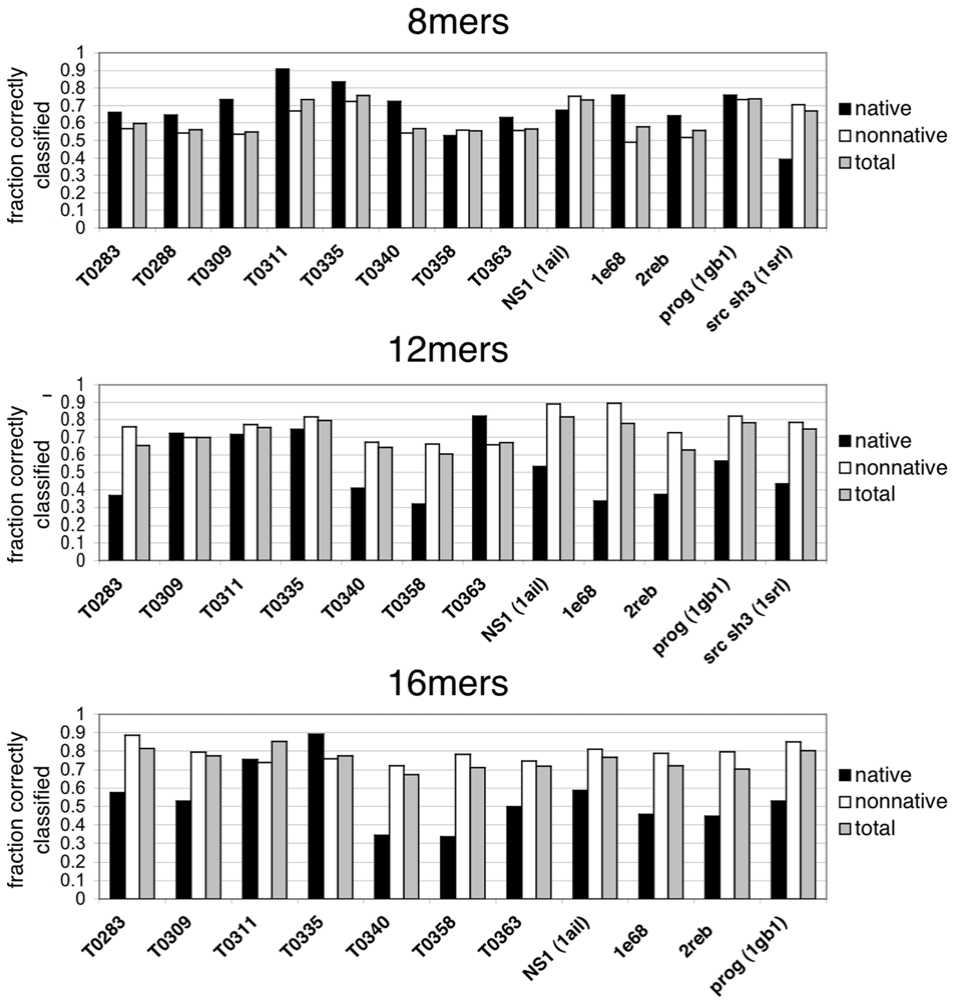
Contact prediction success for all proteins in the test set. Predictions were made using the best logistic regression models built from the 8-mer, 12-mer, and 16-mer simulations.

Now, given the parameters obtained from the logistic-regression models described above, we can compute the probability that a given simulated peptide conformation has native contacts. Figure 5 shows the contact prediction success for all protein targets in the test set. The average percentage of correctly classified contacts (across each protein target) using the 8-mer data is 63.2% (72.3% for native contacts and 60.7% for non-native contacts). The average percentage of correctly classified 12-mer contacts increases to 71.3% (57.3% for native contacts and 74.3% for non-native contacts), and for 16-mer contacts the average classification success is 76.9% (56.3% for native contacts and 80.9% for non-native contacts).

In the case where the data contains many more non-native contacts than native contacts, a high classification accuracy may not reflect a significant improvement over a random null distribution, *per se*. To test this possibility for our selected models, we built a null distribution of contact metrics to test the random-case performance of our models (see Methods). Several statistical tests, including Matthews correlation coefficient (MCC) values and receiver-operator characteristic curves [21] show that our best classification models perform better than random (for a full discussion, see Text S1).

**Figure 6:**
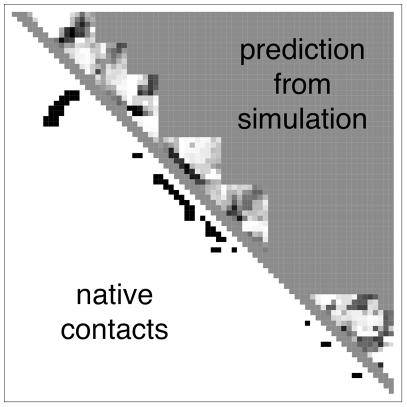
A contact map showing the results of the best 16-mer regression model for an example target, T0363. Above the diagonal, the grayscale values at each contact position correspond to ‘logit’ values 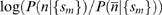 given by the best logistic regression model trained on all the 16-mer simulation data. The background gray value corresponds to contacts not sampled by the fragment simulations, and is colored according to the logit value threshold 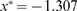 used for the classification criterion; logit values 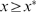 are classified as native and appear darker, while logit values 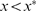 are classified as non-native and appear lighter. On the lower diagonal are shown the native contacts in the range sampled by the fragment simulations. (8-mer, 12-mer, and 16-mer predictions for all targets are shown in Text S1.)

Figure 6 compares the predictions to the true native structures. It shows the ‘logit’ values (see Methods) given by the best 16-mer logistic regression model for an example target. This quantity has the flavor of an informational equivalent of a free energy difference of native minus denatured. The darker black on the figure indicates the strongest prediction of native-like structure. The 8-mer, 12-mer and 16-mer results for all targets is shown in Text S1. Not surprisingly, to the extent that these peptide fragment simulations predict native-like structures, helices are better predicted than hairpins.

**Figure 7:**
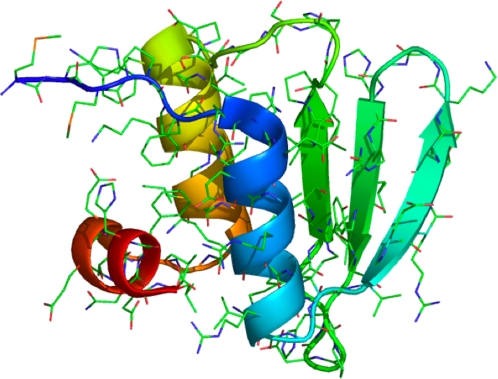
A target from CASP6 (1whz) used to test the classification model. Ribbon diagram of the X-ray crystal structure was made with pymol.

**Figure 8:**
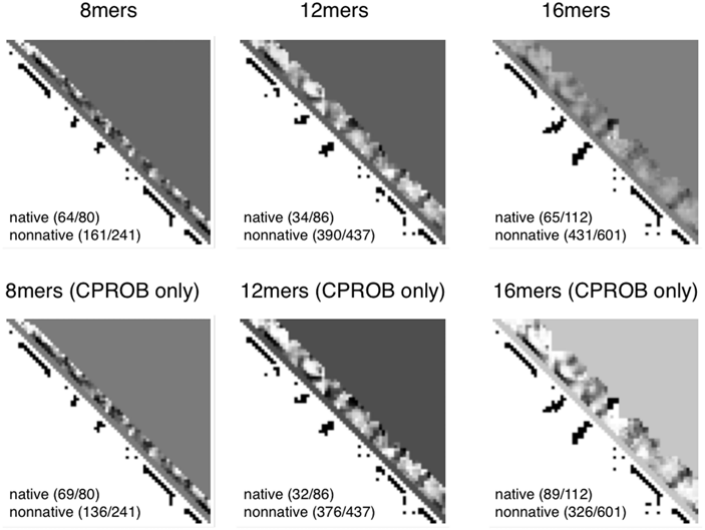
Logit values and prediction successes given by the best classification models for fragment simulations of 1whz. The upper diagonal shows the logit scores 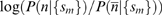 with prediction success rates. The lower diagonal shows native contacts in the range sampled by the fragment simulations. As the fragment simulations increase in length, clear signals of predicted secondary structures begin to emerge. For comparison (bottom row) are shown the logit values and prediction scores given by the best regression model trained only on contact probability. The similarity of the two models shows that most of the predictive power comes directly from the frequency of contacts observed in the simulation data.

Next, we tested our model on a protein outside our test set. We tested 1whz (PDB ID: 1whz), a 70-residue CASP6 target with an 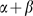 structure taken from *Thermus thermophilus* (Figure 7). REMD simulations of 8-mer, 12-mer, and 16-mer fragments were performed (62, 39, and 74 independent fragment simulations, respectively) using the ZAM procedure, and contact predictions were made using our previously-paramterized 8-mer, 12-mer, and 16-mer logistic regression classification models. Figure 8 shows contact prediction success rates for 1whz, and the logit values for each contact estimated from the 8-mer, 12-mer, and 16-mer data. As the fragment length grows, a consensus resemblance to the native contact map begins to emerge, although incorrect in some places. The logit values are very similar to the logit values given by 8-mer, 12-mer, and 16-mer regression models trained only on contact probability, showing that the contact probability observed in our simulations contains most of the predictive information.

### Extrapolating Inferences from Single Contacts to Larger Structures

These models make per-contact predictions. But, we are interested in predictions for whole peptide conformations. To turn our contact-based scores into conformation-based scores, we compute a score, 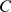, for a given molecular conformation as follows:
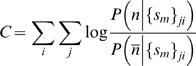
Here, 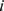 runs over all contacts in the conformation, and 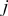 runs over all fragment simulations which contain contact 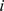.

**Figure 9:**
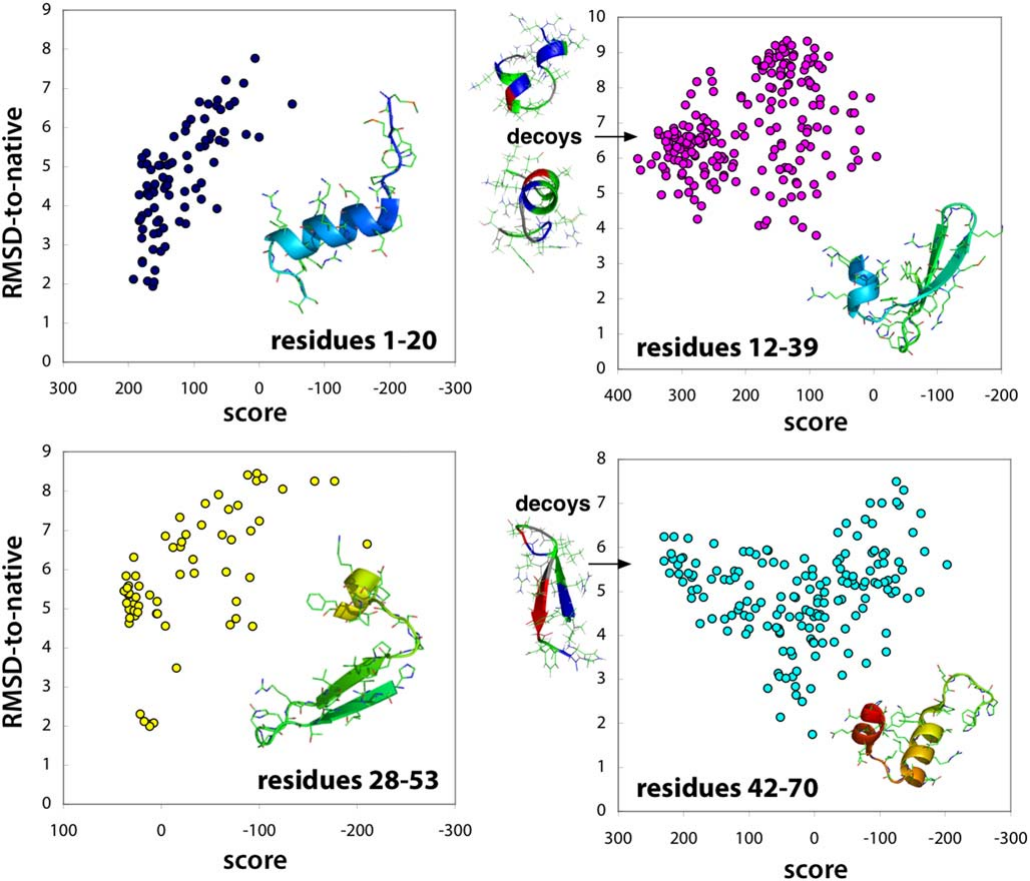
RMSD-to-native of cluster conformations plotted versus cluster conformation scores for all cluster conformations extracted from 16-mer fragment simulations of 1whz. Each dot represents a cluster conformation, color-coded according to its region along the protein sequence: residues 1–20 (cyan), residues 12–39 (magenta), residues 28–53 (yellow), and residues 42–70 (cyan). On the left (residues 1–20 and 28–53) are examples of high conformational cluster scores predicting native structures, while on the right (residues 12–39 and 42–70) are examples of high-scoring decoy structures.

We computed conformation scores for all the cluster conformations extracted from 8-mer, 12-mer, and 16-mer 1whz fragment simulations. For 8-mers and 12-mers, we observe a correlation (albeit noisy) between a high value of 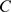 and a near-native (low-RMSD) structure (see Text S1). For 16-mers, the conformation score predicts four likely secondary structures consisting of helices and hairpins along the sequence of the protein (Figure 9). Two of these secondary structures correspond to correctly predicted native structures (the N-terminal helix and C-terminal hairpin), while two of the secondary structures are non-native “decoys.” Even for the decoys, near-native conformations are sampled substantially. Interestingly, the helical decoy seen in the sequence of residues from 12–39 is also predicted by the I-Sites/HMMSTR/Rosetta structure prediction server [22],[23] when templates from multiple sequence alignments are turned off (see Text S1), indicating structural ambivalence.

## Discussion

We have performed computer simulations of short peptides—8-mers, 12-mers and 16-mers—using the AMBER 96 force field and the OBC implicit solvation model. Our aim was to see whether the metastable structures of these fragments bear any resemblance to the conformations those fragments adopt in the native states of the proteins in which they appear. We find that the peptide contact probabilities in a logistic regression model lead to a 76% success rate in 16-mers in correctly classifying contacts as either native or nonnative. Across the chain lengths studied, the false negative rates (native contacts classified as non-native) of our best logistic regression models range from about 30–45%. The false positive rates (non-native contacts classified as native) vary from about 20–40%. These results show these predicted peptide conformations in water are significantly more native-like than would be expected from random conformers. Previously, Bystrof and Garde also showed a 75% success rate at predicting native helicity across 64 8-mer fragments simulated using AMBER ff94 and explicit TIP3P water [14]. This compares with our 72% success rate at classifying native contact for 8-mers. While there remain issues of the accuracy of the forcefield+solvation model [20], and our limited simulation times (5–15 ns), nevertheless, these results indicate that, by using REMD for all-atom sampling and ZAM for conformational searching, small peptide fragments in proteins adopt conformations in solution that significantly resemble the conformations they ultimately adopt in their native proteins. Past experiments have reached similar conclusions, for specific peptide fragments [24].

These results may have useful application in physics-based methods, like ZAM [3],[4] that aim to predict protein structures from all-atom simulations in the absence of knowledge-based secondary structure prediction methods. This work also has implications for understanding how proteins can physically fold up so rapidly to reach their native structures. It suggests that proteins can fold into globally optimal conformations by starting with locally optimal conformations first. While this idea has long been a mainstay of models of protein folding kinetics, this is, as far as we know, the first extensive demonstration in a purely physical model. However, these local propensities alone are not sufficient, at least in our simulations, to predict the native states of proteins.

While our fragment simulations show that some peptide fragments sample native-like states, the sampling still produces many false positives and false negatives. This is consistent with the information-theoretic studies of Crooks and Brenner [25] which examined neural net models trained on local sequence alone, and found that “one fourth of the total information needed to determine secondary structure is available from local inter-sequence correlations.” Similarly, our results also support the idea that cooperative, long-range tertiary contacts are crucial in determining native structure. But while local structuring alone may be insufficient to fold proteins, such information can help to narrow the conformational search. Fleming et al. has shown that while restricting a protein chain to preferred secondary structures *per se* generates random coil-like behavior, some simple additional logic about tertiary cooperativity and hydrogen bonding can predict native-like protein topologies and structures [26]. Moreover, bioinformatics-based protein structure prediction methods have benefitted greatly from fragment assembly methods whereby locally compatible structures dramatically reduce the conformational search problem [22], [27]–[29]. Our results suggest local structural information from physical simulations can improve our understanding of protein folding pathways, and may be useful in physics-based structure prediction.

## Methods

### A Database of Short Protein Fragment Simulations

Our dataset of peptides was 8-mer, 12-mer, and 16-mer fragments of 8 CASP7 target sequences and 5 other protein sequences with known structures taken from the PDB (see Table 2). The 8-mer, 12-mer, and 16-mer fragments cover 100%, 88.7%, and 76.7% of the entire sequence space of the 13 proteins considered, respectively (see also Text S1). We performed computer simulations for 10 ns for each peptide, totaling about 8.7 CPU years in simulation time.

**Table 2:** The 13 test proteins that were used to create a database of simulation fragments (8 CASP7 targets and 5 protein structures from the PDB).

PDB id	CASP target	Name	Residues	Residues in PDB		8-mers	12-mers	16-mers
2hh6	Yes	T0283	112	112	Fragment simulations	36	4	12
2gzv	Yes	T0288	93	93		30	—	—
2h4o	Yes	T0309	76	63		24	7	23
2ict	Yes	T0311	94	94		31	9	32
2hep	Yes	T0335	85	42		13	5	11
2he4	Yes	T0340	90	90		29	16	23
2hjj	Yes	T0358	87	75		28	13	24
2hj1	Yes	T0363	97	87		31	13	23
2reb	No	RecA	60	60		19	6	8
1e68	No	Bacteriocin	70	70		22	21	33
1gb1	No	Protein G	56	56		49	45	21
1ail	No	NS1	70	70		63	37	17
1srl	No	src SH3	56	56		49	45	—
					Total number of contacts	4236	9865	19360
					Simulation replicas	15	15	20
					Total number of simulations	424	221	227
					Simulation time 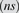	31800	16575	22700
					Total simulation time 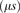	71.1		
					CPU years (10 ns/day)	8.7		

#### Simulation details

We used the AMBER ff96 force field [16] with the solvation model of Onufriev, Bashford, and Case [30] in replica exchange molecular dynamics (REMD) simulations [31]. Each simulation was 5 ns in length, with 15 or 20 replicas (depending on the number needed to achieve a 50% acceptance ratio) ranging from temperatures 270–700 K. Replica swaps between neighboring temperatures were attempted every 20 ps for 8-mers, every 10 ps for 12-mers, and every 5 ps for 16-mers. A set of 10 or less representative conformations, clustered to ∼2Å RMSD by a modified K-means algorithm, is extracted from the data and used for the starting configurations of each next round of simulation.

We simulated the fragments using the ZAM (Zipping and Assembly Method) protocol described in [3],[4]. In the early “growth” stage of ZAM, short molecular dynamics sampling of 8-mer peptide fragments are simulated. The final structures of these simulations are then grown to 12-mers, and further simulated. This continues to the 16-mer stage, at which point several alternative topologies for the cluster conformations extracted from the 12-mer simulations are explored by adding harmonic contact restraints. These restraint energies are later subtracted out when calculating observables from the simulation data using the weighted-histogram analysis method (WHAM) [32]. Contact metrics (see below) were calculated using WHAM at the lowest replica temperature (270 K) from the last nanosecond of the five lowest-temperature replicas (1 ps snapshots).

### Contact Metrics

Classification models were trained on five different contact-based metrics, calculated on a per-contact basis from the simulation data: 1) contact probability (CPROB), 2) a distance profile score (DPROF), 3) a mutual stability score (MSTAB), 4) a mutual cooperativity score (MCOOP) and 5) mesoentropy score (MESO) (Figure 2). These metrics are described in detail below.

#### Contact probability (CPROB) and distance profile score (DPROF)

Contact probability is calculated as the fraction of sampled states that have inter-residue distances less than 8Å (we also tested 7Å, and three different distance definitions; see Training and Testing). The distance profile score (DPROF) was developed to obtain more information about the interaction of two residues as a function of distance, by extracting the potential of mean force 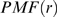 along the contact distance coordinate 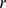. 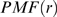 is calculated as 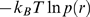 where 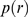 is the observed distribution of contact distances, using the WHAM method. The distance profile score is defined as 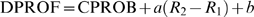, where 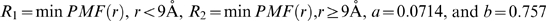. This was a simple heuristic we found by finding the best coefficients 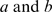 that separated native and non-native contacts in our preliminary tests.

#### Mutual stability score (MSTAB) and mutual cooperatvity score (MCOOP)

These metrics are designed to characterize, for any given contact, the average extent of cooperative (two contact pairs) interactions with the given contact which may indicate (thermodynamic) folding cooperativity.

**Figure 10:**
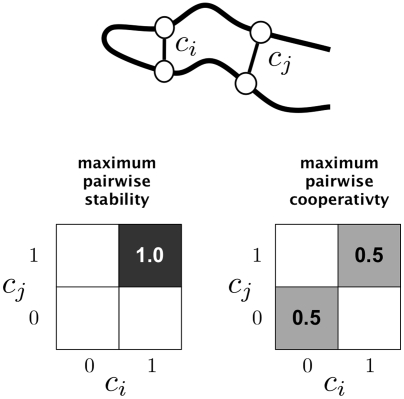
Examples of pairwise stability and pairwise cooperativity used in calculating mutual stability and cooperativity scores. For a particular pair of contacts 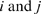, 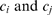 are indicator variables: 1 if the contact is made, and 0 if the contact is not made. The pairwise distribution 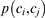 represents the joint probability of contacts 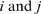 being made or not. Pairwise *stability* is at a maximum when both contacts 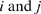 are made with a probability of 1. Pairwise *cooperativity* is maximized when 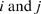 are formed in an all-or-nothing way, so as to maximize the mutual information between 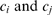.

The mutual stability and cooperativity scores can best be described by considering pairwise distributions of contact probabilities 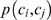, where 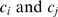 are indicator variables: 0 if the contact is not made, and 1 if the contact is made. We define the *pairwise stability* as 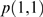, the probability that contacts 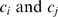 are present simultaneously. The *pairwise cooperativity* is a measure of how interdependent the distributions of 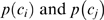 are, defined as the mutual information between variables 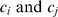:
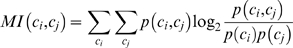
When measured in bits, the pairwise cooperativity is a value between 0 and 1, and provides complementary information to pairwise stability, which also is a value between 0 and 1 (Figure 10). For a given contact 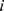, MSTAB is calculated as the number of contact pairs 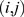 with pairwise stabilities greater than 0.5. Similarly, MCOOP is calculated as the number of contact pairs 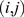 with pairwise cooperativties greater than 0.3 bits.

#### Mesoentropy score (MESO)

The mesoentropy score is related to the backbone entropy. It measures the distribution of backbone dihedral mesostates, defined by Ho and Dill [15] as 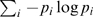, where each 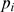 is the probability of a particular mesostate (for example: aabbaballbbb for alpha (a), strand (b) and loop (l) states along a 12-mer fragment). This provides a measure of the conformational diversity of the thermodynamic ensemble at equilibrium. The MESO score is assigned per-contact, but since the mesoentropy is a function of the entire conformational ensemble of a fragment, all contacts in a given fragment simulation receive the same MESO score.

### Bayesian Classification Models

Given the various metrics above, of the peptide conformations observed from the simulations in solution, we now ask if there is a way to combine those metrics to make the best possible predictions of what the peptide's structure is in the native state of the protein. For each contact observed in our database of simulated fragments, we have a set of 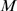 measured contact metrics 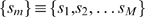, and the known native structure of the fragment, which tells us if the contact is native or non-native. Using this data, we want to train a probabilistic model to estimate the probability of a contact being native versus non-native, given only the contact metrics observed in a peptide simulation. This is a binary pattern classification problem, where we have an unknown parameter 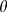 which can be either be native 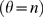 or non-native 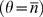, and we wish to calculate 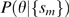. Bayes' formula can be used to restate this posterior probability as
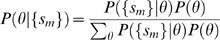
(1)Here, 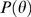 represents our *prior* knowledge of the probability of observing a native or non-native contact, given no other information about that contact. 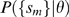 represents the conditional probability of observing a set of metrics 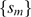 for a contact, given that we know whether that contact is native or non-native.

The ‘naive Bayesian’ approach would be to assume that, for any contact, our set of calculated metrics 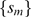 are all mutually independent and uncorrelated. In this case,
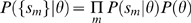
(2)


Using Equations 1 and 2, and taking the logarithm of the ratio of 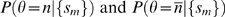, we get
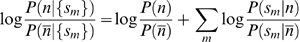
(3)


Since 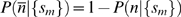, it follows that the log-ratio can be expressed as a linear sum of ‘logit’ terms of the form 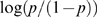. The first term on the right side of Equation 3 is a ‘logit’ for our prior, and the remaining terms are conditional ‘logits’ for our metrics of interests. Both kinds of information are empirically compiled from our database of fragment simulations, from which we extract histogram counts of each metric 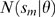 for native and non-native contacts.

Substituting 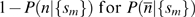, we solve Equation 3 to obtain
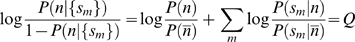
(4)

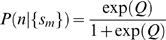
(5)


A potential improvement to the ‘naive Bayes’ model is the *logistic regression* method [33], which seeks to find the best linear coefficients 
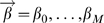
 for the following model:
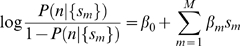
(6)


Solving for 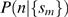 yields
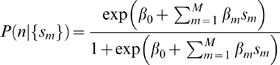
(7)


In practice, these coefficients (and their error estimates) are found with a maximum-likelihood optimization using Newton-Raphson gradient minimization. The optimization is equivalent to least-squared linear regression in the nonlinear ‘logit’ variables 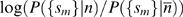. This nonlinearity sometimes makes it possible to obtain better classifications than the naive Bayesian approach.

Note the similarity of the logistic regression model (Equation 6) to the naive Bayesian approach (Equation 4), with 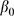 acting as a ‘prior,’ and with the magnitudes of the values of 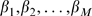 indicating the significance of each contact metric.

### Training and Testing

We built both naive Bayes and logistic regression models for 8-mer, 12-mer, and 16-mer fragments separately. For the naive Bayes models, this involved empirically computing histograms in 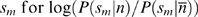. For the logistic regression models, estimates of the best coefficients 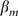 were computed directly from the data, using a freely available Python package [34].

For each of kind of model, in order to determine the best combinations of metrics on which to train the model, we built separate models for all (2^5^−1) = 31 combinations of the five contact metrics (CPROB, DPROF, MSTAB, MCOOP and MESO). In addition, for each of the models, we tested three different inter-residue distance definitions (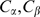, and residue side chain centroid), and two different distance cutoffs to define a contact (7.0Å and 8.0Å), giving a total of 186 combinations to test.

To avoid over-fitting, the training data used to construct each model was divided randomly into five groups so that independent models could be built for each group. Additionally, 1/5 of the data in each group was set aside for testing the model, and the other 4/5 of the data was used to train the model. This means that for each model, there were 25 independent testing and training rounds: 5 independent model-building rounds, each with 5 leave-one-out trials of testing and training.

### Model Selection

To assess which model was the best, we used a statistical hypothesis testing scheme to find a model that most successfully classifies native contacts as well as non-native contacts. Consider a test where we use the statistic 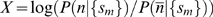 to decide between two hypotheses. The hypothesis 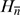 is that the contact is non-native, while the hypothesis 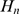 is that the contact is native. If 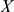 is less than some threshold value 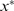, then we accept 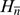 and reject 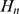, and if 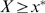, we accept 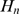 and reject 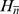. To find the best value for 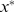, we choose the value that maximizes 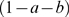, where 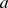 is the fraction of non-native contacts incorrectly classified as native, and 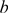 is the fraction of native contacts incorrectly classified as non-native. Even though there are many more non-native contacts than native contacts, this procedure equally weights native and non-native contacts, achieving a balance of specificity and statistical power. We define the *model quality* (Q) as the maximal value of 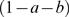, and use the 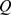 value to rate the relative predictive power of different models. Errors in 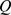 were estimated by examining the sample variance across the five independent trials of the complete model-building procedure.

For the naive Bayes models built for each fragment length, the model that yielded the highest model quality (Q) when applied to testing data was chosen as the best model. For the logistic regression models, the 25 rounds of testing and training produced a series of models across which 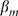 values may correlated. Thus, instead of choosing the average 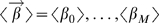 for the best logistic regression model, we chose the model whose coefficients were closest to the centroid of 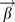 values across the 25 testing and training rounds.

### Contact Prediction Success

For each simulation, the probability of a contact being native can be estimated by Equation 7. However, in the case where there are multiple simulations of the same contact (in overlapping fragment simulations), we can use all of the simulation data to estimate this probability. Assuming that each of 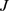 simulations is statistically independent, the probability of a particular contact being native is estimated by:
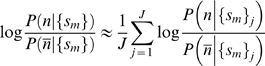
We use these combined estimates of 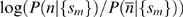 with the original hypothesis testing cutoffs 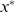 to classify contacts as native or non-native. The percentage of contacts correctly classified this way is what we report as our contact prediction success rate.

### Calculation of Null Distributions

A null distribution in C-alpha RMSD values for 8-mers, 12-mers and 16-mers was calculated by taking 10000 random pairwise samples of 8-mer, 12-mer and 16-mer fragments from a set of 3465 protein structures taken from the SCOP database [35] (1 structure, or 2 if existing, from each unique SCOP class).

Because there are correlations between contact metrics due to chain connectivity, considerable care was taken to construct null distributions for contact metrics that preserved these correlations. We did this by constructing the null distribution on a fragment-by-fragment basis. For each fragment, the values of the contact metrics were retained, while the assignment of native and non-native contacts was randomized according to a per-fragment bootstrapping procedure. For each fragment, a random contact map was drawn (with replacement) from the full data set. This reassignment procedure, across the entire set of fragments, was repeated 1000 times to construct a distribution of random-case realizations.

## Supporting Information

Text S1 Supplemental Data and Results
(8.84 MB PDF)

## References

[1] Simons K, Kooperberg C, Huang E, Baker D (1997). Assembly of protein tertiary structures from fragments with similar local sequences using simulated annealing and bayesian scoring functions.. J Mol Biol.

[2] Rohl C, Strauss C, Misura K, Baker D (2004). Protein structure prediction using rosetta.. Methods Enzymol.

[3] Ozkan SB, Wu GH, Chodera JD, Dill KA (2007). Protein folding by zipping and assembly.. Proceedings of the National Academy of Sciences.

[4] Shell MS, Ozkan SB, Voelz V, Wu GA, Dill KA (2008). A blind test of physics-based prediction of protein structures.. Biophysical Journal.

[5] Voelz VA, Dill KA (2007). Exploring zipping and assembly as a folding principle.. Proteins: Structure, Function, and Bioinformatics.

[6] Gong H, Rose GD (2005). Does secondary structure determine tertiary structure in proteins?. Proteins.

[7] Scott KA, Alonso DOV, Pan Y, Daggett V (2006). Importance of context in protein folding: secondary structural propensities versus tertiary contact-assisted secondary structure formation.. Biochemistry.

[8] Baldwin RL, Rose GD (1999). Is protein folding hierarchic? i. local structure and peptide folding.. Trends Biochem Sci.

[9] Daggett V, Fersht AR (2003). Is there a unifying mechanism for protein folding?. Trends Biochem Sci.

[10] Jones DT (1999). Protein secondary structure prediction based on position-specific scoring matrices.. J Mol Biol.

[11] Snow C, Sorin E, Rhee Y, Pande V (2005). How well can simulation predict protein folding kinetics and thermodynamics?. Annu Rev Biophys Biomol Struct.

[12] Price DJ, Brooks CL (2002). Modern protein force fields behave comparably in molecular dynamics simulations.. Journal of Computational Chemistry.

[13] Shell MS, Ritterson R, Dill KA (2008). A test on peptide stability of amber force fields with implicit solvation.. The Journal of Physical Chemistry B.

[14] Bystroff C, Garde S (2003). Helix propensities of short peptides: molecular dynamics versus bioinformatics.. Proteins.

[15] Ho BK, Dill KA (2006). Folding very short peptides using molecular dynamics.. PLoS Computational Biology.

[16] Cornell WD, Cieplak P, Bayly CI, Gould IR, Kenneth M, Merz J (1995). A second generation force field for the simulation of proteins, nucleic acids, and organic molecules.. Journal of the American Chemical Society.

[17] Onufriev A, Bashford D, Case DA (2004). Exploring protein native states and large-scale conformational changes with a modified generalized born model.. Proteins.

[18] Frishman D, Argos P (1995). Knowledge-based protein secondary structure assignment.. PROTEINS: Structure, Function, and Genetics.

[19] Scholtz JM, Baldwin RL (1992). The mechanism of alpha-helix formation by peptides.. Annual Review of Biophysics and Biomolecular Structure.

[20] Roe DR, Okur A, Wickstrom L, Hornak V, Simmerling C (2007). Secondary structure bias in generalized born solvent models: comparison of conformational ensembles and free energy of solvent polarization from explicit and implicit solvation.. The Journal of Physical Chemistry B, Condensed matter, materials, surfaces, interfaces biophysical.

[21] Baldi P, Brunak S, Chauvin Y, Andersen C (2000). Assessing the accuracy of prediction algorithms for classification: an overview.. Bioinformatics.

[22] Bystroff C, Shao Y (2002). Fully automated ab initio protein structure prediction using i-sites, hmmstr and rosetta.. Bioinformatics.

[23] Bystroff C, Thorsson V, Baker D (2000). Hmmstr: a hidden markov model for local sequence-structure correlations in proteins.. Journal of Molecular Biology.

[24] Blanco FJ, Rivas G, Serrano L (1994). A short linear peptide that folds into a native stable beta-hairpin in aqueous solution.. Nat Struct Biol.

[25] Crooks GE (2004). Protein secondary structure: entropy, correlations and prediction.. Bioinformatics.

[26] Fleming PJ, Gong H, Rose GD (2006). Secondary structure determines protein topology.. Protein Sci.

[27] Bujnicki JM (2006). Protein-structure prediction by recombination of fragments.. Chembiochem.

[28] Chikenji G, Fujitsuka Y, Takada S (2006). Shaping up the protein folding funnel by local interaction: lesson from a structure prediction study.. Proc Natl Acad Sci U S A.

[29] Kim DE, Chivian D, Baker D (2004). Protein structure prediction and analysis using the robetta server.. Nucleic Acids Research.

[30] Onufriev A, Bashford D, Case DA (2004). Exploring protein native states and large-scale conformational changes with a modified generalized born model.. PROTEINS: Structure, Function, and Bioinformatics.

[31] Sugita Y, Okamoto Y (1999). Replica-exchange molecular dynamics method for protein folding.. Chemical Physics Letters.

[32] Kumar S, Bouzid D, Swendsen RH, Kollman PA, Rosenberg JM (1992). The weighted histogram analysis method for free-energy calculations on biomolecules. i. the method.. Journal of Computational Chemistry.

[33] Hastie T, Tibshirani R, Friedman J (2001). The Elements of Statistical Learning.

[34] Whitaker J (2006). Python packages.. http://www.cdc.noaa.gov/people/jeffrey.s.whitaker.

[35] Lo Conte L, Ailey B, Hubbard TJP, Brenner SE, Murzin AG (2000). Scop: a structural classification of proteins database.. Nucleic Acids Research.

