# Supplementary material for: Predicting Peptide Structures in Native Proteins from Physical Simulations of Fragments
Source: PLoS Comput Biol. 2009 Feb 6;5(2):e1000281. doi: 10.1371/journal.pcbi.1000281 (PMC2629132; doi:10.1371/journal.pcbi.1000281)
Supplement: Text S1 — Supplemental Data and Results (8.84 MB PDF) [file pcbi.1000281.s001.pdf]

Predicting peptide structures in native proteins from physical simulations SUPPLEMENTAL DATA

Vincent A. Voelz, M. Scott Shell and Ken Dill

Contents

1 Sequence coverage of fragment simulations 2

2 Classification success for the best logistic regression models 5

3 Testing the performance of the best logistic regression models against random null distributions 6

4 Regression models trained on local and non-local contacts only 14

5 Benchmark simulations of 16-mer fragments 17

6 Contact Prediction success for 8-mer and 12-mer fragment simulations 22

7 Conformation scores for 8-mer and 12-mer fragment simualtions 22

8 An examination of decoy structures for 1whz 23

9 Acknowledgments 26

Preprint submitted to PLoS Compbio *22 November 2008*

1 Sequence coverage of fragment simulations

The 8-mer, 12-mer, and 16-mer fragment simulations cover 100%, 88.7%, and 76.7% of the entire sequence space of the 13 proteins considered, respectively. Sequence spans for 8-mers, 12-mers and 16-mers are shown in Figures 1, 2 and 3.

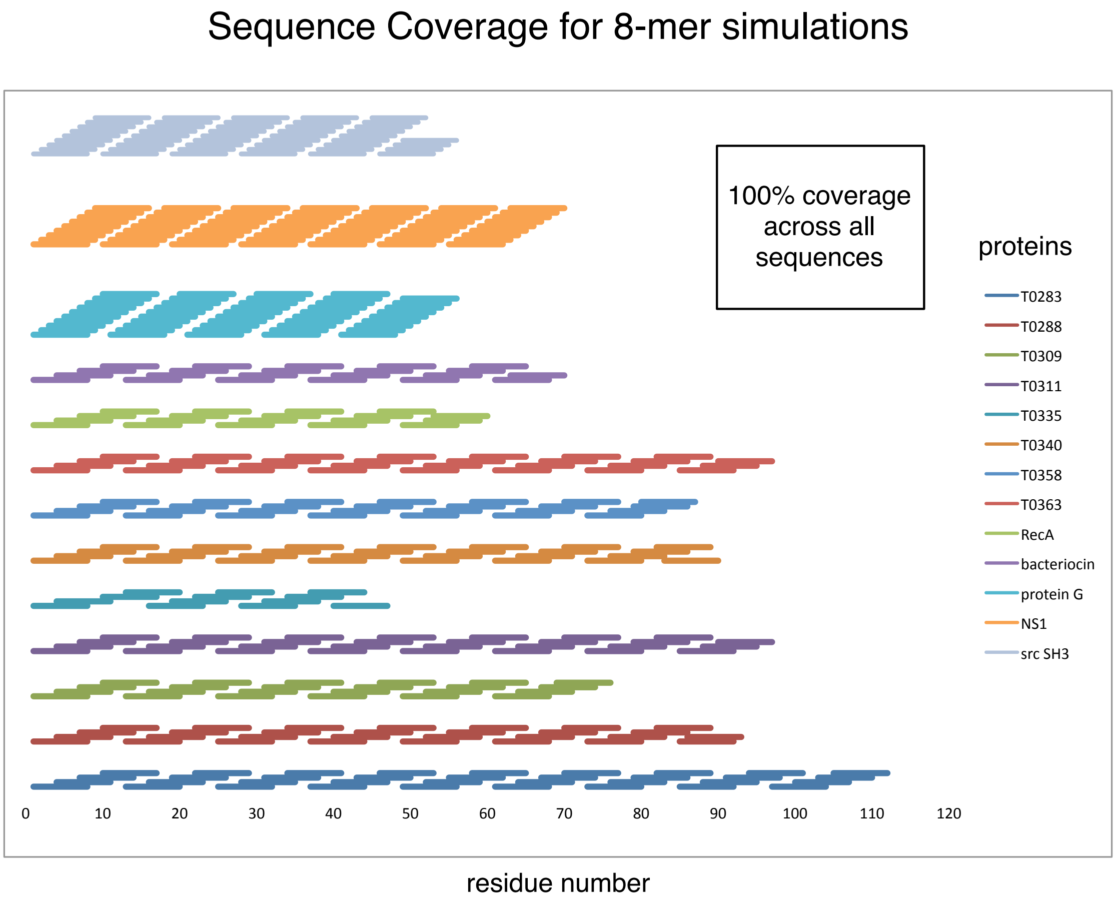

Fig. 1. 8-mer fragment simulations cover 100% of the sequence of the 13 proteins studied.

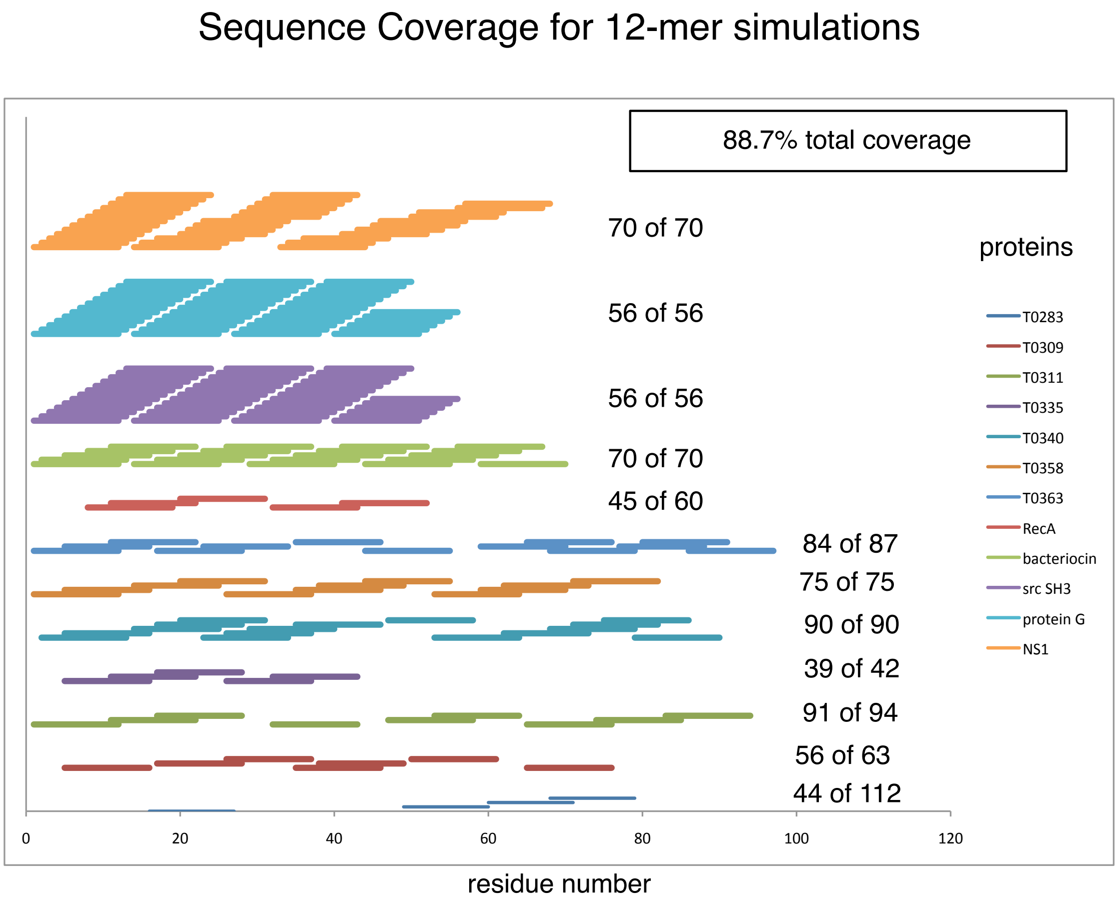

Fig. 2. 12-mer fragment simulations cover 88.7% of the sequence of the 13 proteins studied.

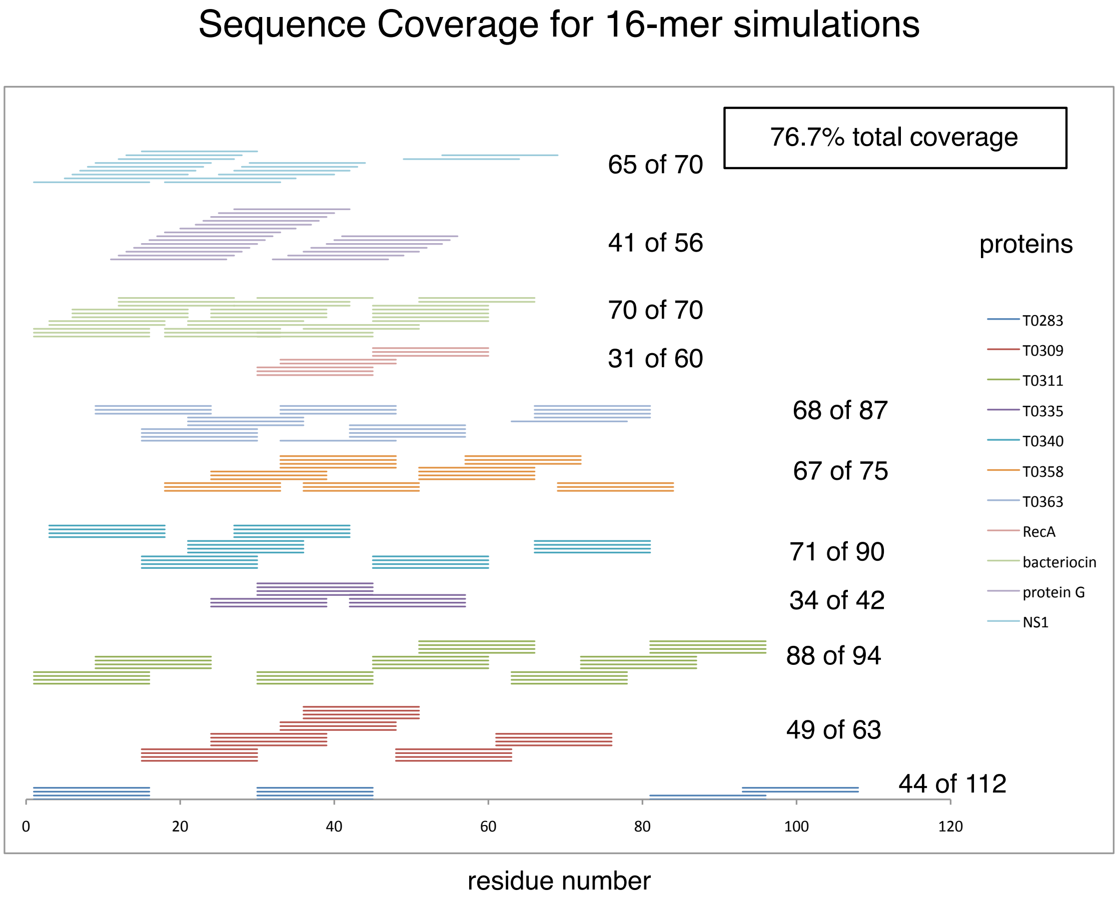

Fig. 3. 16-mer fragment simulations cover 76.7% of the sequence of the 12 proteins studied (src SH3 was not included in the 16-mer simulations). Fragments with the same sequence span represent simulations with competing sets of harmonic contact restraints.

2 Classification success for the best logistic regression models

Table 1 shows the success of classifying native and non-native contacts given by our best-parameterized logistic regression models. The best classification successes of models with fewer included terms are also shown, for comparison.

Table 1
Classification success for the best logistic regression models.

length	dist. method	included terms	model quality (Q)	x^*	native classification success	non-native classification success
8	C_α	CPROB	.2095 ± .0025	-1.1688	0.7055 (436/618)	0.6015 (1437/2389)
		+MSTAB	.2225 ± .0021	-1.1688	0.7055 (433/618)	0.6023 (1439/2389)
12	C_β	CPROB	.2220 ± .0010	-1.346	0.6012 (466/775)	0.7270 (2791/3839)
		+MSTAB	.2318 ± .0025	-1.2382	0.5522 (428/775)	0.7392 (2838/3839)
		+MESO	.2427 ± .0005	-1.2056	0.5135 (398/775)	0.7619 (2925/3839)
16	C_α	CPROB	.2526 ± .0005	-1.4704	0.6439 (539/837)	0.7072 (3096/4278)
		+DPROF	.2617 ± .0005	-1.520	0.6678 (559/837)	0.7034 (3010/4278)
		+MSTAB	.2690 ± .0007	-1.353	0.5926 (496/837)	0.7667 (3280/4278)
		+MCOOP	.2702 ± .0006	-1.307	0.5675 (475/837)	0.7840 (3354/4278)

3 Testing the performance of the best logistic regression models against random null distributions

Recall the model-selection procedure we used: To judge the quality of each model, we used an accuracy-based measure, $Q = 1 - a - b$, where a is the fraction of incorrectly-classified native contacts, and b is the fraction of incorrectly-classified non-native contacts. The Q quantity was used to forward-select models with increasing numbers of logistic regression parameters.

There is complicating issue when using accuracy-based measures to select models. In the case where the data contains many more non-native contacts than native contacts (or vice versa), a high classification accuracy may not reflect a significant improvement over a random null distribution, *per se*. To test this possibility for our selected models, we built a null distribution of contact metrics to test the random-case performance of our models.

Because there are correlations between contact metrics due to chain connectivity, considerable care was taken to construct null distributions for contact metrics that preserved these correlations. We did this by constructing the null distribution on a fragment-by-fragment basis. For each fragment, the values of the contact metrics were retained, while the assignment of native and non-native contacts was randomized according to a per-fragment bootstrapping procedure. For each fragment, a random contact map was drawn (with replacement) from the full data set. This reassignment procedure, across the entire set of fragments, was repeated 1000 times to construct a distribution of random-case realizations.

We next performed several tests to compare the performance of our selected models on the actual data versus the random null data, which we will describe below.

Figures 4, 5, 6, 7, 8 and 9, show the results of these tests for the classification models chosen as the best (based on model quality). Figures 4 and 5 show the results for our best 8-mer and 12-mer classification models, respectively. Figures 6, 7, 8 and 9 show the results for a series of best 16-mer classification models, having one, two, three, and four regression terms, respectively. The model with four regression terms was chosen as the the best according to the model quality, Q.

Part (A) of each figure shows the classification success for native contacts given by the model, for both random null data and the actual data. Part (B) of the figures shows the same, for non-native contacts. The green line shows a distribution of classification successes, across all 1000 realizations of null set data, whereas the blue line simply marks the classification success achieved when the classification model is applied to the actual data. In general, non-native contacts are predicted with more significance than native contacts, a trend which increases with chain length and the number of metrics used to train the logistic regression model. This trend is due (at least in part) to the fact that the ratio of non-native to native contacts increases with chain length.

Part (C) of each figure shows a contour plot of the joint distribution of native and non-

native classification successes, across all 1000 realizations of null set data. The star marks the classification successes when the model is applied to actual data. The contour plot is an interpolation made using the program *Mathematica*. In all cases, the p-values for joint classification success is less than 0.001 (which is the limit of what we can say given 1000 null realizations).

Part (D) of each figure shows the model quality, Q, for the selected models across a range of classification thresholds. The blue line shows the model quality when applied to the actual data. The green line shows the average model quality across the 1000 random null realizations, as a function of classification threshold. The error bars above and below the green line show the standard deviation across random null realizations, for each classification threshold.

Part (E) of each figure is similar in form to Part (D), but instead shows the Matthews Correlation Coefficient (MCC) as a function of classification threshold (1). The MCC is a quantity used to characterize the quality of a binary classification from the full "confusion matrix" containing the numbers of true positives (TP), false positives (FP), true negatives (TN) and false negatives (FN). It is been shown to be a more balanced measure of classification accuracy when the classes are of very different size. The MCC is defined as:

$$MCC = \frac{TP \times TN - FP \times FN}{\sqrt{(TP+FP)(TP+FN)(TN+FP)(TN+FN)}}$$

The results shown in parts (D) and (E) show that our chosen regression models have classification accuracies better than random. It is interesting to note that the models are somewhat predictive even when applied to our random null data. This may be partly due to the mismatch in the numbers of native and non-native contacts, but it may also be due to correlations present in the data independent of sequence. For instance, native contacts in general have small sequence separation, which may correlate with the propensity of a random polymer chain to make local contacts more frequently.

Part (F) of each graph shows a graph of the true positive rate versus the false positive rate as the classification threshold is varied. This is otherwise known as a receiver operating characteristic (ROC) curve (1). In the case where a model has no power to discriminate whatsoever between native and non-native contacts, the ROC curve is a straight diagonal, shown for reference as a red line. The more discriminative power a model has, the more the ROC curve should be off the diagonal, which is what we see in this graph. For all models, the ROC curve for the best classification models applied to the actual data (blue) is farther from the diagonal than the ROC curve for the random null data (green). The null data ROC curve shown is the average across all 1000 null distribution realizations.

Part (G) of each graph shows, for reference, the number of true positives (TP), false positives (FP), true negatives (TN) and false negatives (FN) for each classification model, applied to the true set of actual contact metrics. (Note that the total number of contact metrics

contained in the set may be larger than the number of contacts in the full set of proteins, because the set includes contact metrics from all the fragment simulations, many of which contain overlapping protein sequences).

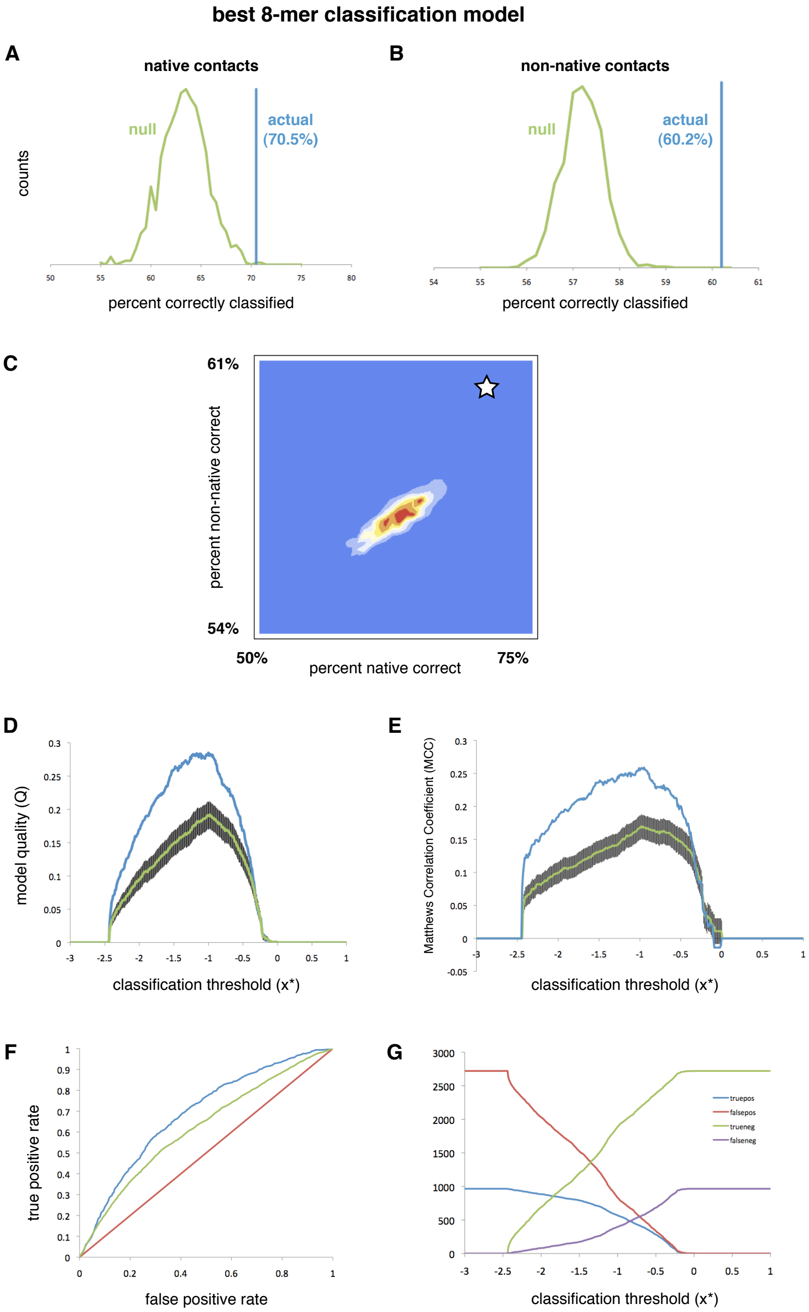

Fig. 4. The performance of the best 8-mer classification model on actual data versus random null data (see description above for details).

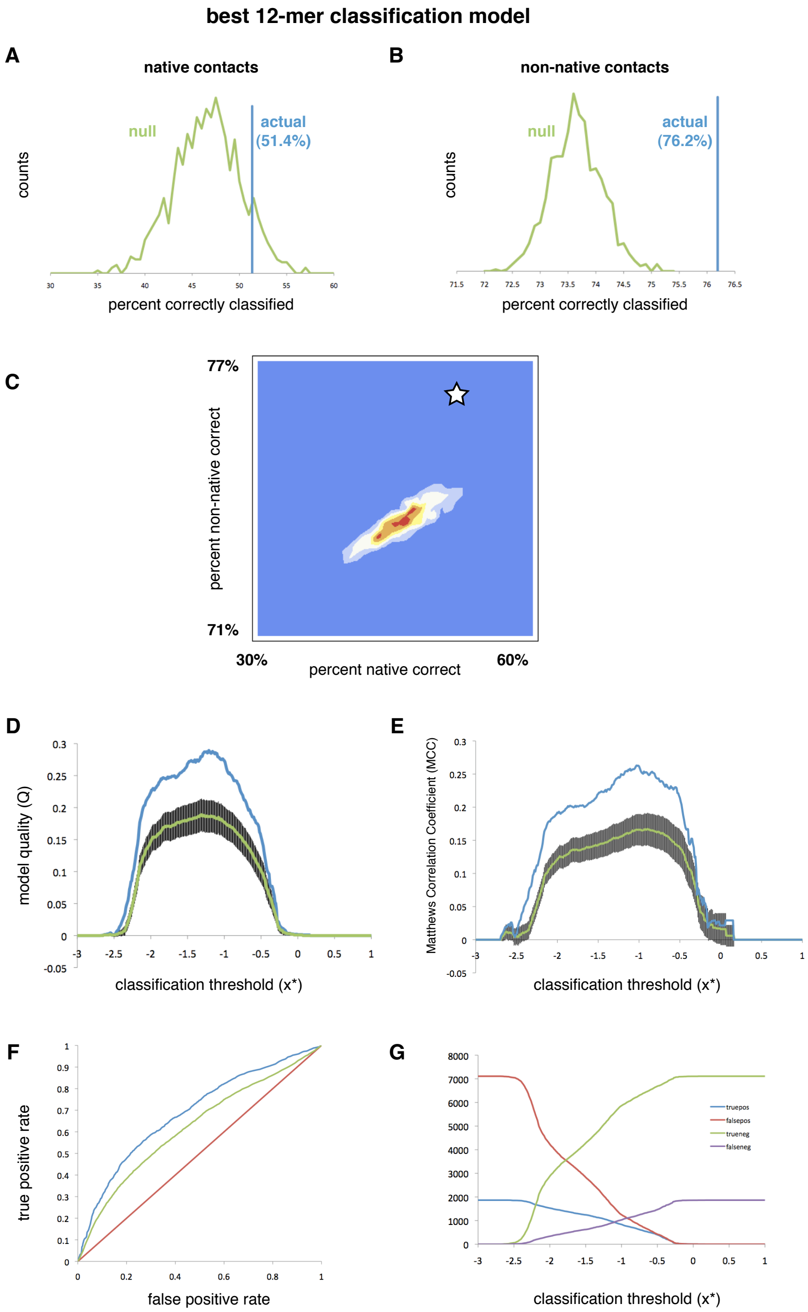

Fig. 5. The performance of the best 12-mer classification model on actual data versus random null data (see description above for details).

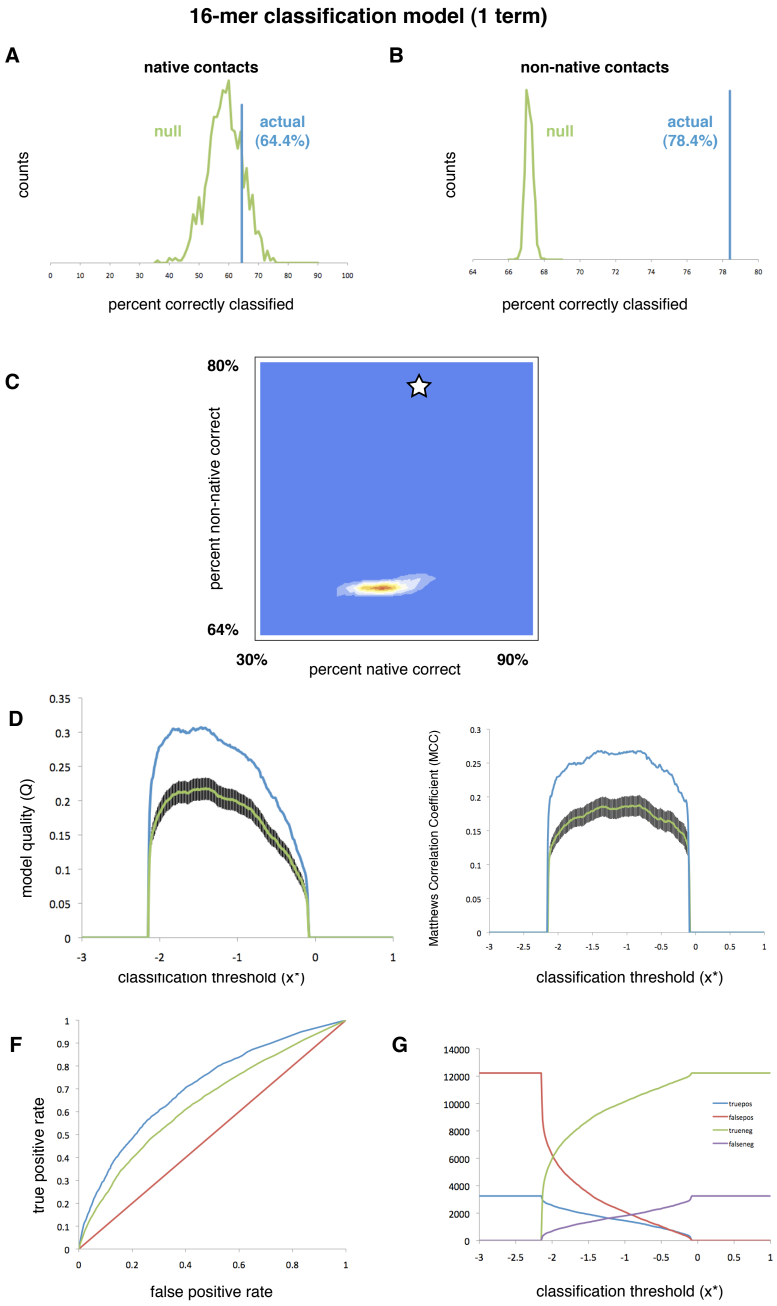

Fig. 6. The performance of the best one-term 16-mer classification model on actual data versus random null data (see description above for details).

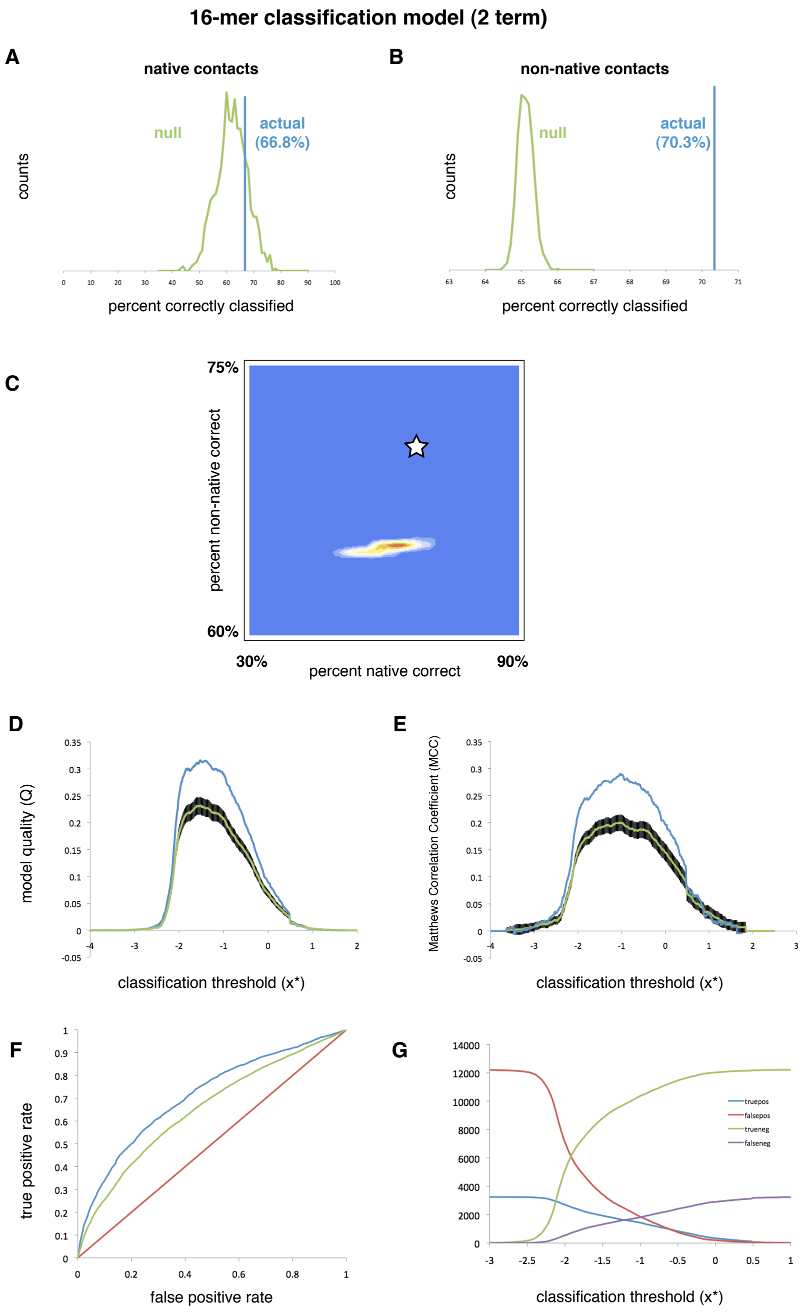

Fig. 7. The performance of the best two-term 16-mer classification model on actual data versus random null data (see description above for details).

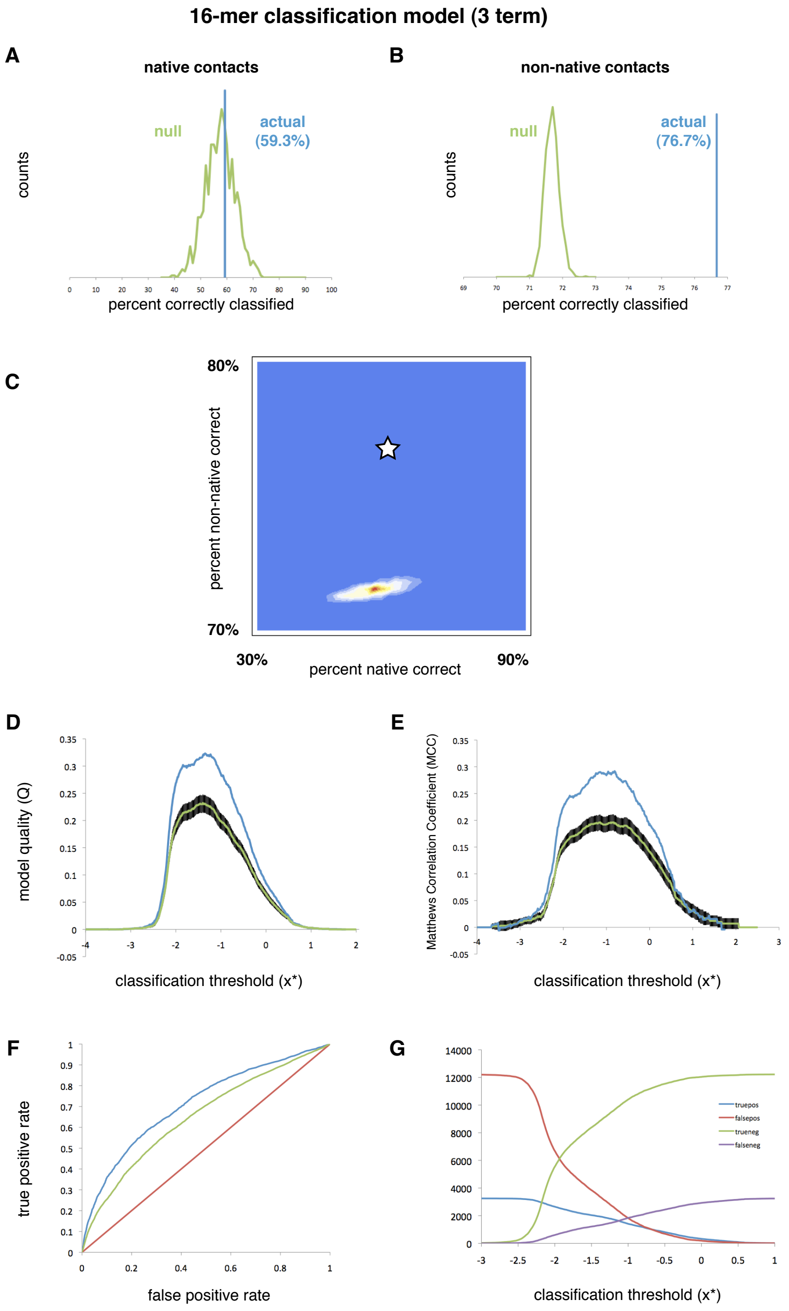

Fig. 8. The performance of the best three-term 16-mer classification model on actual data versus random null data (see description above for details).

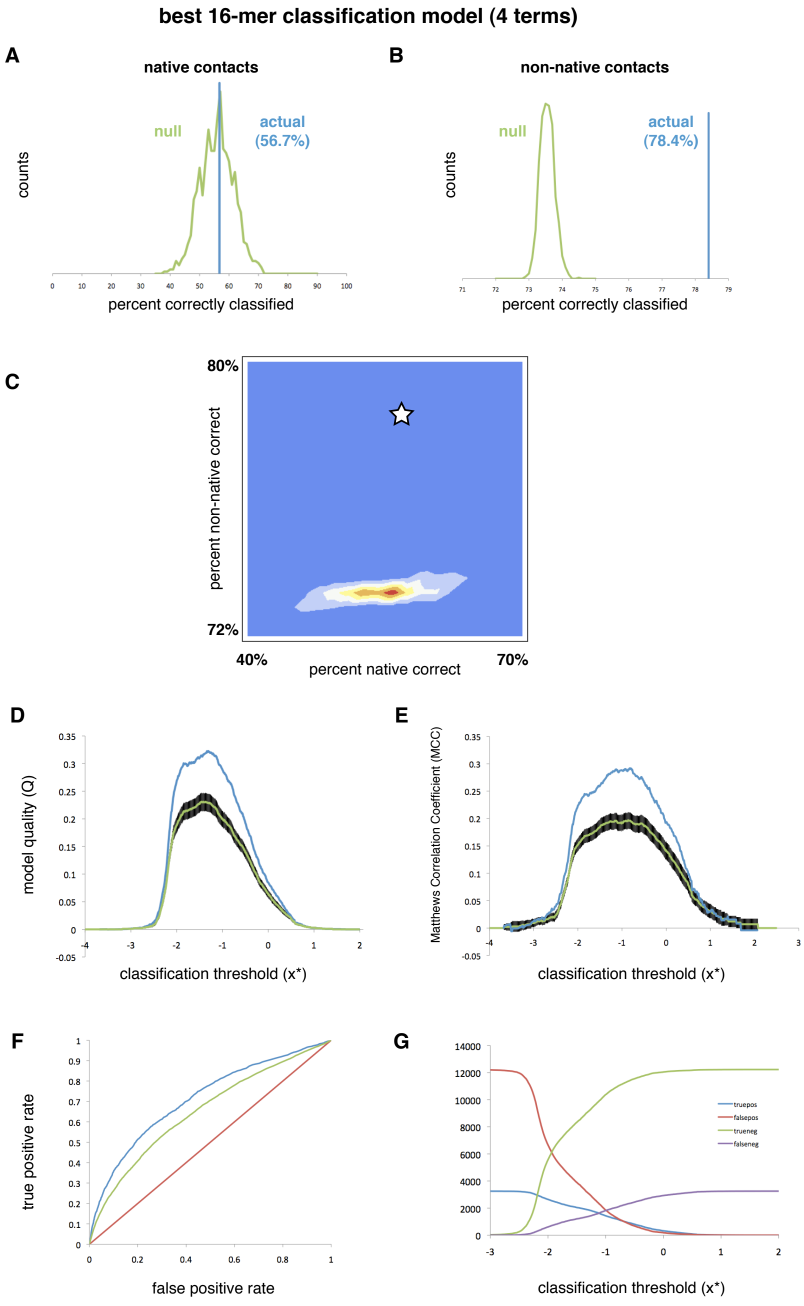

Fig. 9. The performance of the best (four-term) 16-mer classification model on actual data versus random null data (see description above for details).

4 Regression models trained on local and non-local contacts only

Can we achieve better logistic regression models to classify contacts as native or non-native, if we train separately on the data for local contacts ($|i - j| \leq 4$) and non-local contacts ($|i - j| \geq 5$)? The training data was divided into two groups, one with contacts having a sequence separation of 3 or 4 residues, and another with sequence separations of 5 residues or more. The best logistic regressions for the local and nonlocal data are shown in Tables 2 and 3, respectively, and the model relevances (R) for each metric are shown in Figures 10, respectively.

Overall, the classification success for the local-only or nonlocal-only data was comparable, but never as high as the classification success using the combined data. Contact probabilities remain the most important metric in the local-only and nonlocal-only regressions, although for local contacts, the model relevance (R) values for the CPROB metric was smaller in the 8-mer and 12-mer regression models. This is probably because local contacts are easily sampled by the chain regardless of sequence, which adds noise to the classification problem.

Table 2
Classification success for the best logistic regression models trained only on local contacts (sequence separations of 3 or 4 residues only).

length	dist. method	included terms	model quality (Q)	x^*	native classification success	non-native classification success
8	C_α	CPROB	.2062 ± .0053	-0.23	0.6974 (431/618)	0.5939 (1419/2389)
12	C_β	CPROB	.2225 ± .0021	-0.45	0.5793 (449/775)	0.7345 (2820/3839)
		+DPROF	.2276 ± .0028	-0.45	0.5187 (402/775)	0.7626 (2928/4278)
16	C_β	CPROB	.2480 ± .0009	-0.33	0.6431 (483/751)	0.7245 (3162/4364)
		+MESO	.2660 ± .0015	-0.249	0.6133 (449/732)	0.7638 (3348/4383)
		+MSTAB	.2684 ± .0013	-0.253	0.6202 (454/732)	0.7554 (3311/4383)

Table 3
Classification success for the best logistic regression models trained only on nonlocal contacts (sequence separations of 5 residues or greater).

length	dist. method	included terms	model quality (Q)	x^*	native classification success	non-native classification success
8	C_α	CPROB	.2074 ± .0036	-2.20	0.688 (432/618)	0.609 (1455/2389)
		+MSTAB	.2092 ± .0044	-2.10	0.686 (424/618)	0.6299 (1505/2389)
12	C_β	CPROB	.2204 ± .0010	-2.31	0.593 (460/775)	0.731 (2806/3839)
		+MSTAB	.2302 ± .0021	-2.26	0.579 (449/775)	0.713 (2738/3839)
16	C_α	CPROB	.2493 ± .0022	-2.70	0.647 (542/837)	0.723 (3095/4278)
		+DPROF	.2565 ± .0021	-2.67	0.629 (527/837)	0.742 (3177/4278)

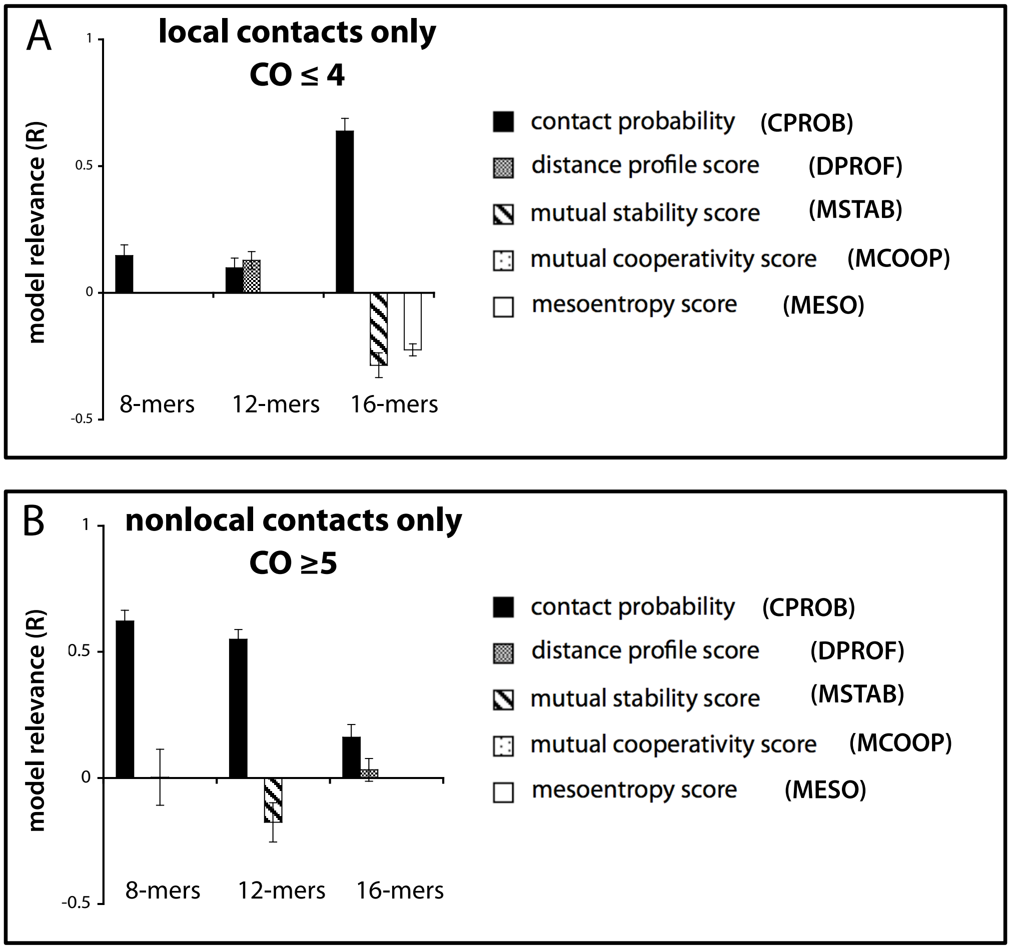

Fig. 10. (A) Model relevances (R) for contact metrics in logistic regression models trained on local contact data only. (B) Model relevances (R) for contact metrics trained on nonlocal contact data only.

5 Benchmark simulations of 16-mer fragments

One of the challenges in the ZAM protocol (2) is sampling a diverse population of conformations using short fragment simulations. To explore different possible topologies, harmonic contact restraints are added to 16-mer simulations to bias the sampling. At this stage, it is particularly critical to sample beta-hairpin structures, which generally take longer to form in short simulations because of their nonlocal topology.

Here, as a test of our sampling methodology, we simulate a set of peptide fragments shown to sample beta-hairpins during the course of the ZAM algorithm for up to 100 ns. Additionally, we simulate 16-mer fragments of a protein outside our training set (PDB code: 1whz), with several different combinations of contact restraints.

There are two issues we try to address with these simulations. The first is to test whether convergence can be achieved with longer simulation times, and if so, whether this can better discriminate between native and non-native hairpin decoys. We find that longer simulation times do not necessarily result in better convergence to native structures, validating our use short fragment simulations. This also suggests that the accuracy of our simulation results is limited more by our forcefield potential than by sampling.

The second issue we address is to test whether the harmonic restraints added at the 16-mer stage can yield better sampling of hairpins, and if this is the case, whether hairpin restraints overly bias the sampling. By comparing across simulations with different sets of restraints, we find that harmonic restraints can help to sample a diversity of conformations in short REMD simulations, and that our prediction results are robust to perturbations from the restraint potentials.

Methods

Molecular dynamics simulation.

The AMBER ff96 force field (3) with the solvation model of Onufriev, Bashford, and Case (4) was used to perform replica exchange molecular dynamics (REMD) simulations (5). Each simulation was 100 ns in length, with ranging from temperatures 270-700K. Clustering was done to both reduce the amount of data to process, and to generate good representatives of conformational basins predicted by the forcefield. A set of 10 or less representative conformations, clustered to ∼2Å RMSD by a modified K-means algorithm, is extracted from the (lowest-temperature) data and used for the starting configurations of future rounds of simulation.

The ZAM (Zipping and Assembly Method) protocol for simulating fragments is as previous described in (2; 6). In the early “growth” stage of ZAM, we rely on short molecular dynamics

sampling of 8-mer peptide fragments which are then grown to 12-mers for further simulation. At the 16-mer stage, several alternative topologies for the cluster conformations extracted from the 12-mer simulations are explored by adding harmonic contact restraints (with a force constant of 0.5 kcal/mol/$\AA^2$) to the residue sidechain centroids.

ZAM simulations of 16-mer hairpins.

20 simulations of beta-hairpin systems were performed for 8 peptide fragments taken from 7 proteins, representing 40,000 ns of total simulation, or about 11 CPU-years (assuming 10 ns/day per processor). A summary of the fragments whose native states are hairpins is described in Table 4. A summary of the "decoy" fragments, whose native states are not hairpins, is described in Table 5. The native structures of the 8 fragments simulated here are shown in Figure 11. The decoys are either amphipathic helices or helix-turn-helix motifs.

Table 4
16-mer ZAM simulations of fragments which are hairpins in the native state.

simulation	protein	residues	sequence	restraints
1	protein G	41-56	GEWTYDDATKTFTVTE	none
2				(Y45,F52)
3	T0363	15-30	IEIAYAFPERYYLKSF	none
4				(A18,Y25)
5				(A20,Y25)
6	T0340	21-36	LHSDKSRPGQYIRSVD	none
7				(P28,I32)
8				(S26,I32)
9				(S26,Y31)

Table 5
16-mer ZAM simulations of "decoy" fragments which are not hairpins in the native state.

simulation	protein	residues	sequence	restraints
10	T0283	30-45	EYHHAYKAIQKYMWTS	none
11				(I38,Y41)
12				(Y35,Y41)
13	T0311	30-45	MEIAPSTASRLLTGK	none
14				(T37,R40)
15	1e68	6-21	GIPAAVAGTVLNVVEA	none
16				(A12,V15)
17				(V11,L16)
18		30-45	SILTAVGSGGLSLLAAA	none
19				(G36,G39)
20				(V35,L40)

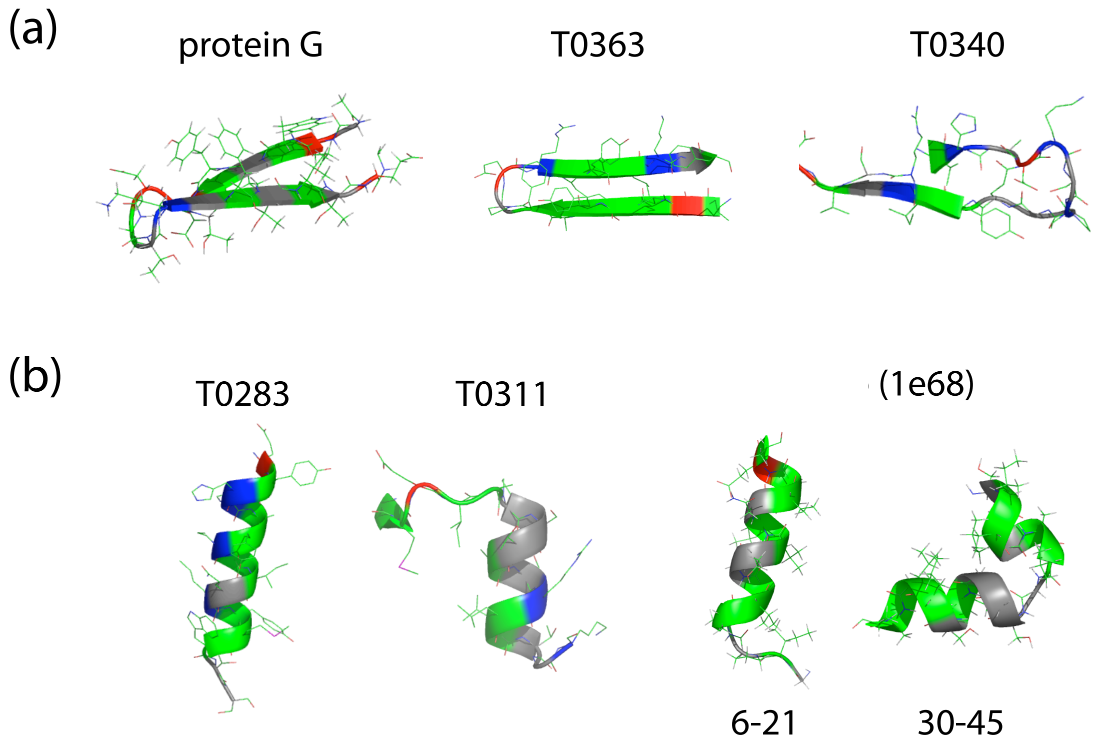

Fig. 11. (a) Protein fragments that are beta-hairpins in their native conformations: residues 41-56 of protein G (1gb1), residues 15-30 of T0363 (CASP7 target) , and residues 21-36 of T0340 (CASP7 target). (b) Protein fragments that have previously been shown to sample hairpin "decoy" structures in short (5 ns) REMD simulations: residues 30-45 of T0283 (CASP7 target), residues 30-45 of T0311 (CASP7 target) , and residues 6-21 and 30-45 of protein 1e68. The ribbon colors denote hydrophobic residues (Ala, Met, Phe, Leu, Cys, Ile, Val, Trp, Tyr) in green, acidic resides (Glu, Asp) in red, and basic residues (Lys, Arg) in blue.

Results

Here we briefly summarize the main findings observed in our hairpins simulations. We note that most of these features are also observed in our test set of peptide fragment simulations.

Figures 19 through 38 show the snapshots of conformations sampled over time for each fragment simulation. The coloring convention is the same as in Figure 11. Each graph shows the conformation clusters and their populations in increments of 10 ns, for the lowest temperature of the simulations. The last nanosecond of each 10 ns-increment was used to generate the conformation clusters. shown in the plot. Note that in many cases, these structures appear to fluctuate quite a bit. This is due to a combination of the intrinsic conformational fluctuations seen in any one replica, and the temperature-swapping done in replica exchange molecular dynamics (REMD).

1. Native hairpin structures are better sampled when the fragment is centered around the beta-turn.

This may be because hairpins out of the larger tertiary context will not tolerate unpaired ends, and instead prefer to make either ionic (salt-bridge) interactions, or hydrogen-bonded states like helices. This effect may be further magnified by the implicit solvent model used, which favors compact states. The T0340 simulations, having the native turn slightly off-center in the sequence, are a good example of this. The conformations sampled in the first 10 ns of restrained and unrestrained simulations are loosely defined by side chain interactions, although some beta-hairpins are sampled with high probability in simulations with (S26,I32) restrained.

2. An $(i, i+5)$ restraint works well to bias the sampling toward beta-hairpins.

Shown in Figure 12 are contact maps showing contact probabilities across all 16-mer simulations, grouped by the sequence separation of restrained residues. It is interesting to note that similar conditional probabilities of observing hairpins is observed in the statistics of native protein structures. From a set of 3465 protein structures taken randomly the SCOP database (7) (1 structure, or 2 if existing, from each unique SCOP class), we computed the conditional probabilities of contacts in protein structures, given that an $(i, i+5)$ contact was already present. The results show a high conditional probability of hairpin-like conformations, regardless of sequence. This is consistent with previous work in polymer models showing how this effect can arise from intrachain excluded volume (8).

Restraints help in sampling beta-hairpins at early times.

Simulations of the C-terminal fragment of protein G, whose native structure is ∼40% hairpin in solution (9), are about 8% hairpin after 10 ns in unrestrained simulations (Figure 19), and mostly hairpins when restrained by (Y45,F52) (Figure 20). Simulations of the T0283 (decoy) fragment also show restraints encourage hairpin formation after 10 ns (Figures 28, 30 and 29). T0311 fragment simulations (Figures 31 and 32) and 1e68 fragment simulations (Figures 33, 34, 35, 36, 37, 38) sample hairpins regardless of restraints, while other fragment simulations are more ambiguous (Figures 21, 22, 23).

Do longer simulation times help fragments converge to native-like structures?.

In general, no. There are several reasons why this may be the case: (1) simulations longer than the 100 ns performed here would be needed, (2) the physical model we used is not perfect, or (3) tertiary context is needed to drive them into their native states. While our simulations do not address the tertiary context, we do observe anecdotal evidence of both (1) and (2). Of course, without complete convergence, it is difficult to assess the quality of our forcefield, other than by the results of our predictions (described in the main text).

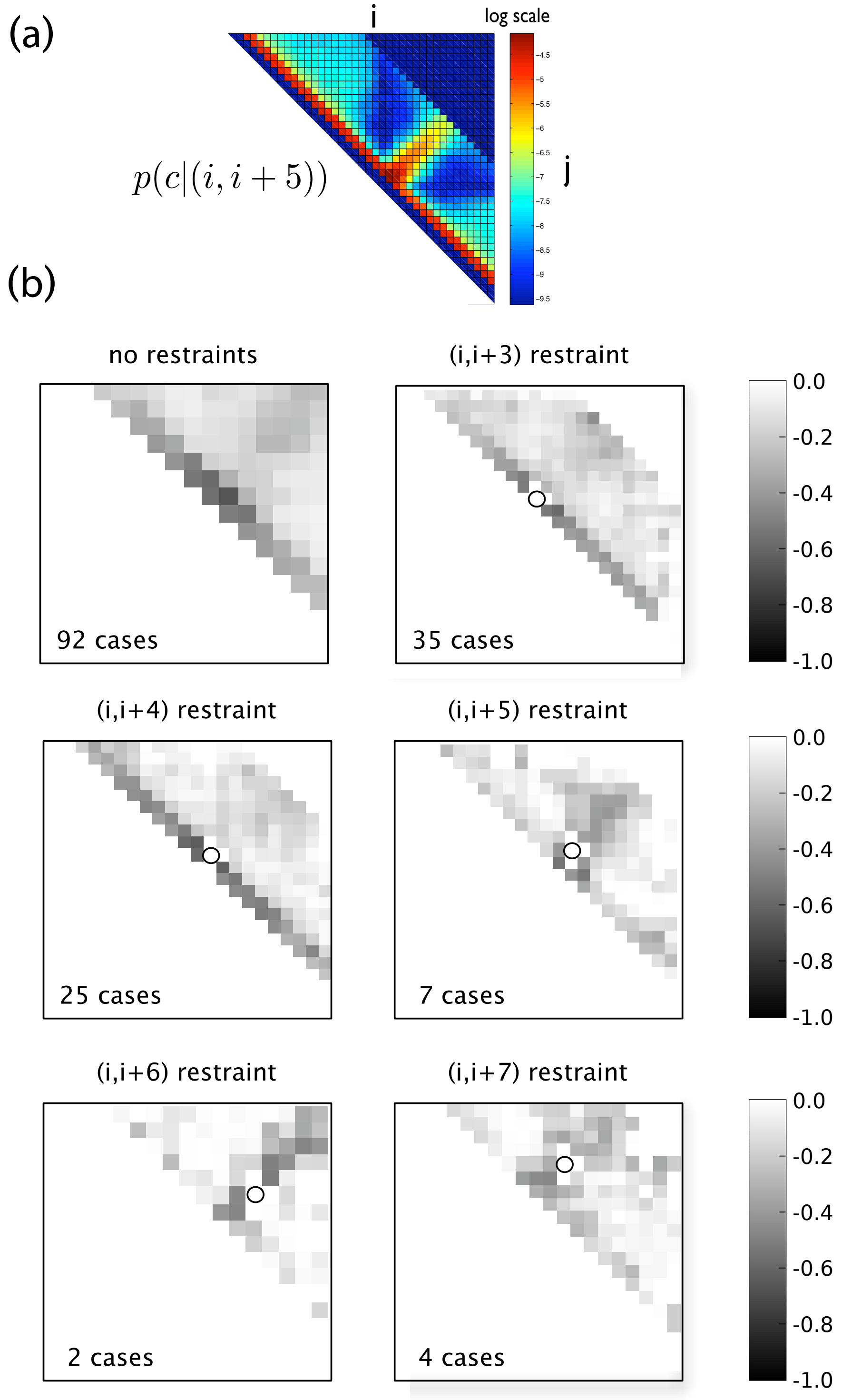

Fig. 12. (a) Conditional probabilities of $C_\alpha(< 7\mathring{A})$ contacts, given an $(i, i+5)$ contact is already present, compiled from 3465 protein structures randomly chosen from the SCOP database (1 structure, or 2 if existing, from each unique SCOP class). (b) Average contact probabilities for ZAM fragment simulations with contact restraints of contact order 3,4,5,6 and 7, aligned on contact map according to their contact restraints, shown by a circle on each contact map. These results were compiled from the database of fragment simulations described in the main text.

For many of our hairpin simulations, there exists quite a bit of variability in the lowest-temperature conformations, and/or population shifts on time scales comparable to the length of the simulation. The simulation results also have features consistent with well-known inaccuracies of Generalized Born models (10; 11**?**), including possible over-stabilization of helices for hairpin fragments with known native states (Figure 19), and some problems with overly stable salt-bridge formation.

Overall, the variability of conformations sampled in these simulations at early times, along with the directed sampling achieved with contact restraints (Figures 20, 22, 23), is further validation that 10 ns of REMD simulation is sufficient for obtaining good sampling.

16-mer fragment simulations are robust to restraint perturbations.

To test whether contact prediction scores are robust with respect to restraints, we compared the results of our usual ZAM protocol of using restrained REMD simulation against 1) the results of simulations that had no restraints, and 2) the results of simulations with competing sets of restraints which were allowed to exchange between replicas. We find that these different protocols have little effect on the outcome of the simulations, as shown by the similarity in prediction scores (Figure 13).

6 Contact Prediction success for 8-mer and 12-mer fragment simulations

Figures 15, 16 and 16 show on a contact map, for each protein target, the 'logit' values of $\log(P(n|\{s_m\})/P(\bar{n}|\{s_m\}))$ given by the best 8-mer, 12-mer and 16-mer logistic regression models for all proteins in our test set. This quantity has the flavor of an informational equivalent of a free energy difference of native minus denatured. The darker black on the figure indicates the strongest prediction of native-like structure.

7 Conformation scores for 8-mer and 12-mer fragment simualtions

We compute a conformation score, C, for a given molecular conformation as follows:

$$C = \sum_i \sum_j \log \frac{P(n|\{s_m\}_{ji})}{P(\bar{n}|\{s_m\}_{ji})}$$

Here, i runs over all contacts in the conformation, and j runs over all fragment simulations which contain contact i.

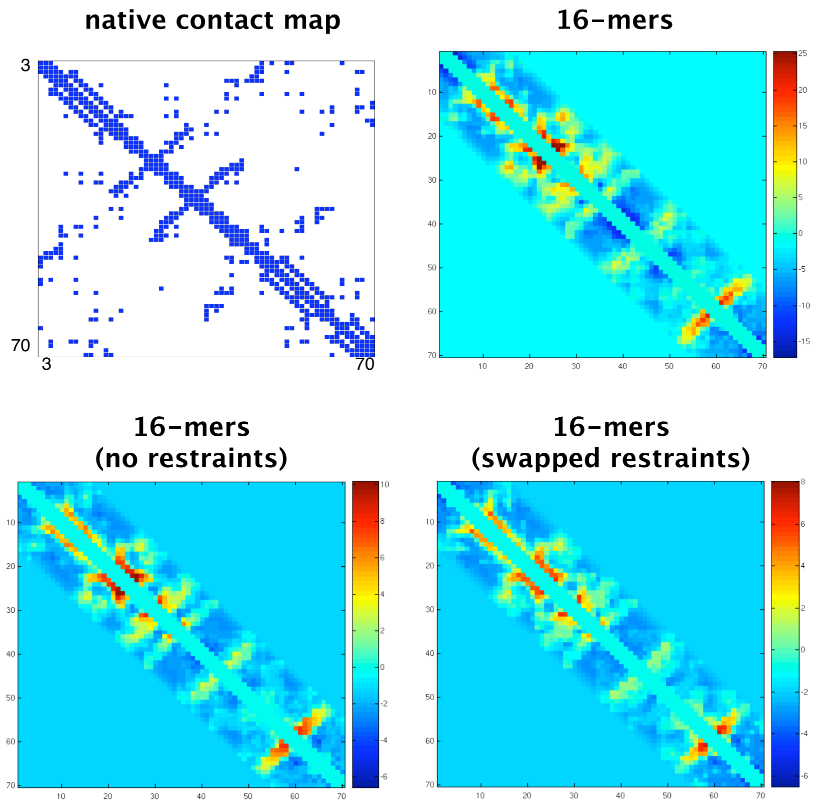

Fig. 13. Contact prediction scores $\sum_j \log(P(n|\{s_m\}_j)/P(\bar{n}|\{s_m\}_j)$ for 16mer fragment simulations of 1whz. (A) Native contact map for the protein 1whz. (B) Results of simulations using the usual ZAM protocol restrained REMD simulation. (C) Results of simulations that had no restraints. (D) Results of simulations with competing sets of restraints which were allowed to exchange between replicas.

We computed conformation scores for all the cluster conformations extracted from 8-mer, 12-mer, and 16-mer 1whz fragment simulations. For 8-mers and 12-mers, we observe a correlation (albeit noisy) between a high value of C and a near-native (low-RMSD) structure. Figure 14 shows the RMSD-to-native plotted versus prediction scores for all cluster conformations extracted from 8-mer and 12-mer fragment simulations of 1whz. The plots are reasonably funnel-shaped, that is, the higher the prediction score, the closer that conformation is to the native state.

8 An examination of decoy structures for 1whz

Recall that for our test protein 1whz, non-native “decoy” conformations were found that gave high native conformation scores. We compared these conformations to the sequence-based predictions from I-sites, a library of local sequence-structure correlations (12). Local I-sites predictions with the highest confidence scores often have corresponding fragment

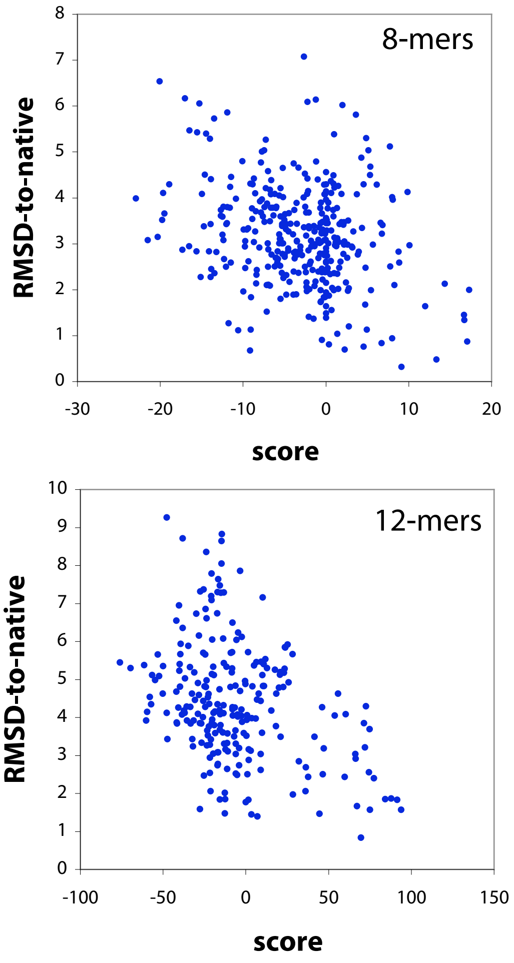

Fig. 14. RMSD-to-native plotted versus prediction scores for all cluster conformations extracted from 8-mer and 12-mer fragment simulations of 1whz.

structures that are experimentally stable in solution. It has been shown that I-sites fragments can be used as ‘initiation sites’ to generate a set of decoy structures that can then be further optimized for protein structure prediction (13; 14). This idea has been used to build an automated protein structure prediction server (14) which uses a hidden Markov model based on I-sites, called HMMSTR, combined with optimization from the Robetta algorithm (15; 16; 17).

When the 1whz sequence is submitted to the HMMSTR/Robetta structure prediction server (14) (with an option set to avoid the use of multiple sequence alignments), the N-terminal beta-strand that our fragment simulations predict as a helical decoy is also predicted to be helical, with a high confidence score of 0.85 (Figure 18). When multiple sequence alignment is used, this helical I-sites fragment receives much lower confidence scores. This observation is further evidence that statistical occurrences of peptides in structural databases are related to their physical free energies.

References

1. Baldi P, Brunak S, Chauvin Y, Andersen C (2000) Assessing the accuracy of prediction algorithms for classification: an overview. Bioinformatics 16:412–424.
2. Ozkan SB, Wu GH, Chodera JD, Dill KA (2007) Protein folding by zipping and assembly. Proceedings of the National Academy of Sciences 104:11987–11992.
3. Cornell WD, Cieplak P, Bayly CI, Gould IR, Kenneth M Merz J, et al. (1995) A second generation force field for the simulation of proteins, nucleic acids, and organic molecules. Journal of the American Chemical Society 117:5179–5197.
4. Onufriev A, Bashford D, Case DA (2004) Exploring protein native states and large-scale conformational changes with a modified generalized born model. PROTEINS: Structure, Function, and Bioinformatics 55:383–394.
5. Sugita Y, Okamoto Y (1999) Replica-exchange molecular dynamics method for protein folding. Chemical Physics Letters 314:141–151.
6. Shell MS, Ozkan SB, Voelz VA, Wu GH, Dill K (2007) A blind test of the zipping and assembly method for *ab initio* protein structure prediction. submitted .
7. Lo Conte L, Ailey B, Hubbard TJP, Brenner SE, Murzin AG, et al. (2000) Scop: a structural classification of proteins database. Nucleic Acids Research 28:257–259.
8. Chan HS, Dill KA (1989) Intrachain loops in polymers: Effects of excluded volume. Journal of Chemical Physics 90:492–509.
9. Blanco FJ, Serrano L (1995) Folding of protein g b1 domain studied by the conformational characterization of fragments comprising its secondary structure elements. Eur J Biochem 230:634–49.
10. Roe DR, Okur A, Wickstrom L, Hornak V, Simmerling C (2007) Secondary structure bias in generalized born solvent models: comparison of conformational ensembles and free energy of solvent polarization from explicit and implicit solvation. The journal of physical chemistry B, Condensed matter, materials, surfaces, interfaces biophysical 111:1846–57. doi:10.1021/jp066831u.
11. Geney R, Layten M, Gomperts R, Hornak V, Simmerling C (2006) Investigation of salt bridge stability in a generalized born solvent model. Journal of Chemical Theory and Computation 2:115–127.
12. Bystroff C, Baker D (1998) Prediction of local structure in proteins using a library os sequence-structure motifs. Journal of Molecular Biology 281:565–577.
13. Bystroff C, Thorsson V, Baker D (2000) Hmmstr: a hidden markov model for local sequence-structure correlations in proteins. Journal of Molecular Biology 301:173–190.
14. Bystroff C, Shao Y (2002) Fully automated ab initio protein structure prediction using i-sites, hmmstr and rosetta. Bioinformatics 18:S54–S61.
15. Chivian D, Kim DE, Malmström L, Bradley P, Robertson T, et al. (2003) Automated prediction of casp-5 structures using the robetta server. PROTEINS: Structure, Function, and Genetics 53:524–533.
16. Kim DE, Chivian D, Baker D (2004) Protein structure prediction and analysis using the robetta server. Nucleic Acids Research 32:W526–W531.
17. Chivian D, Kim DE, Malmström L, Schonbrun J, Rohl CA, et al. (2005) Prediction of casp6 structures using automated robetta protocols. PROTEINS: Structure, Function, and Bioinformatics 7:157–166.

9 Acknowledgments

We thank Fred Davis for his help in compiling the contact statistics from the SCOP database.

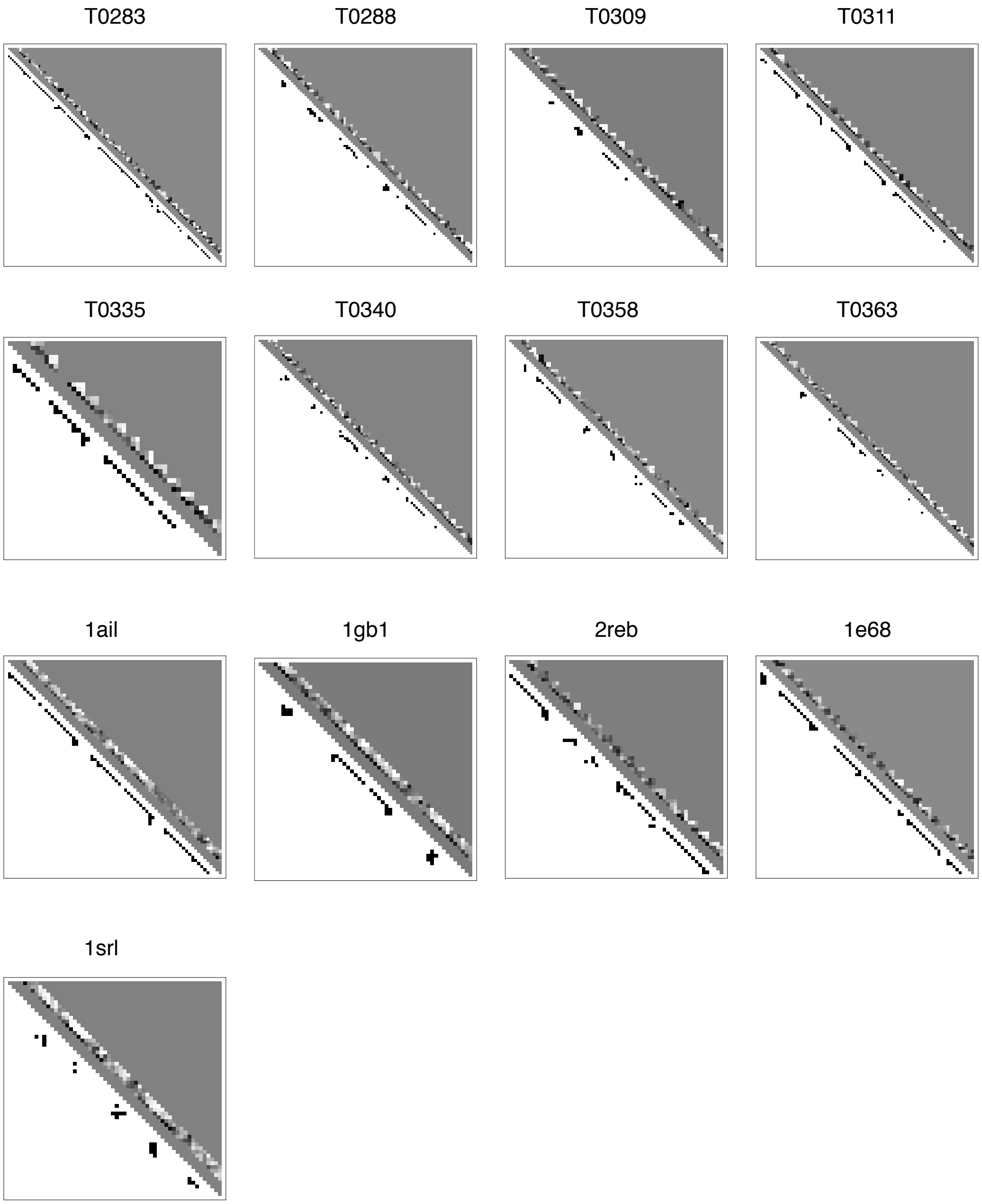

Fig. 15. Contact prediction 'logit' scores of the best regression models trained on 8-mer simulations, for all proteins in our test set. Shading is as described in Figure 5 of the main text.

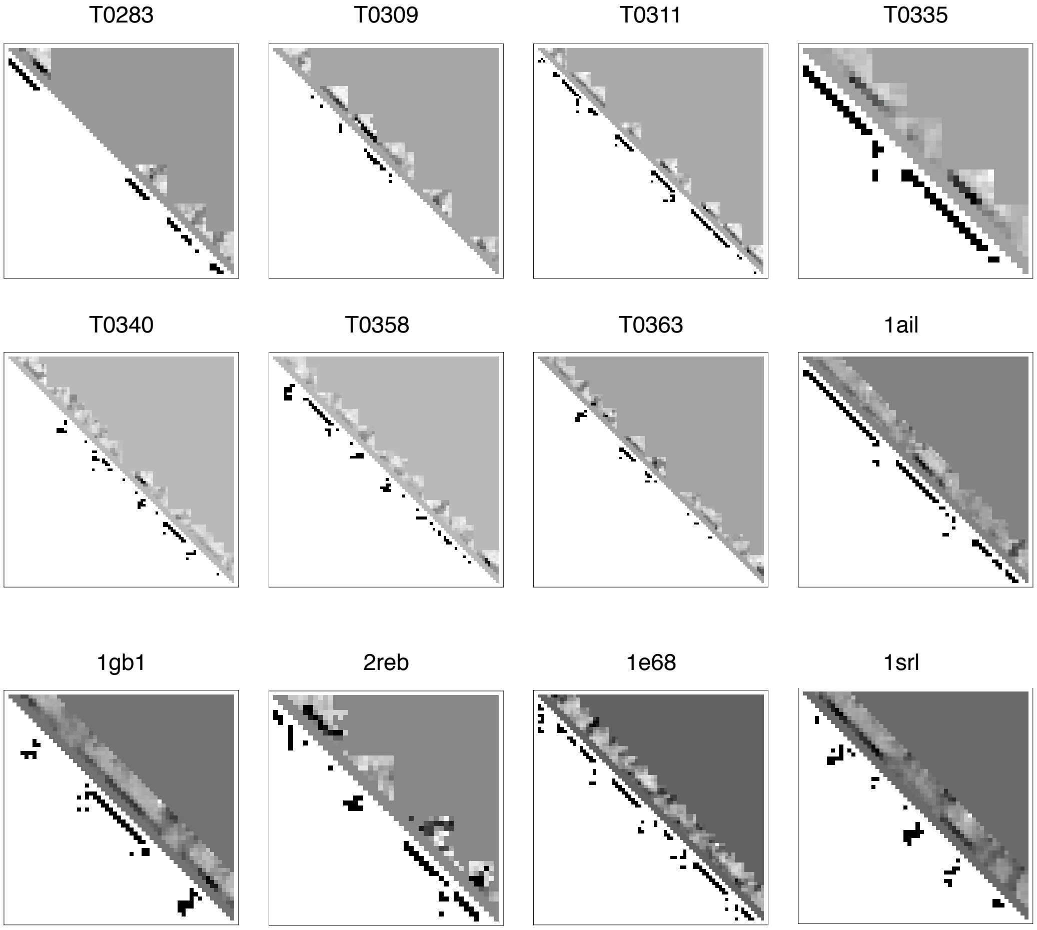

Fig. 16. Contact prediction 'logit' scores of the best regression models trained on 12-mer simulations, for all proteins in our test set. Shading is as described in Figure 5 of the main text.

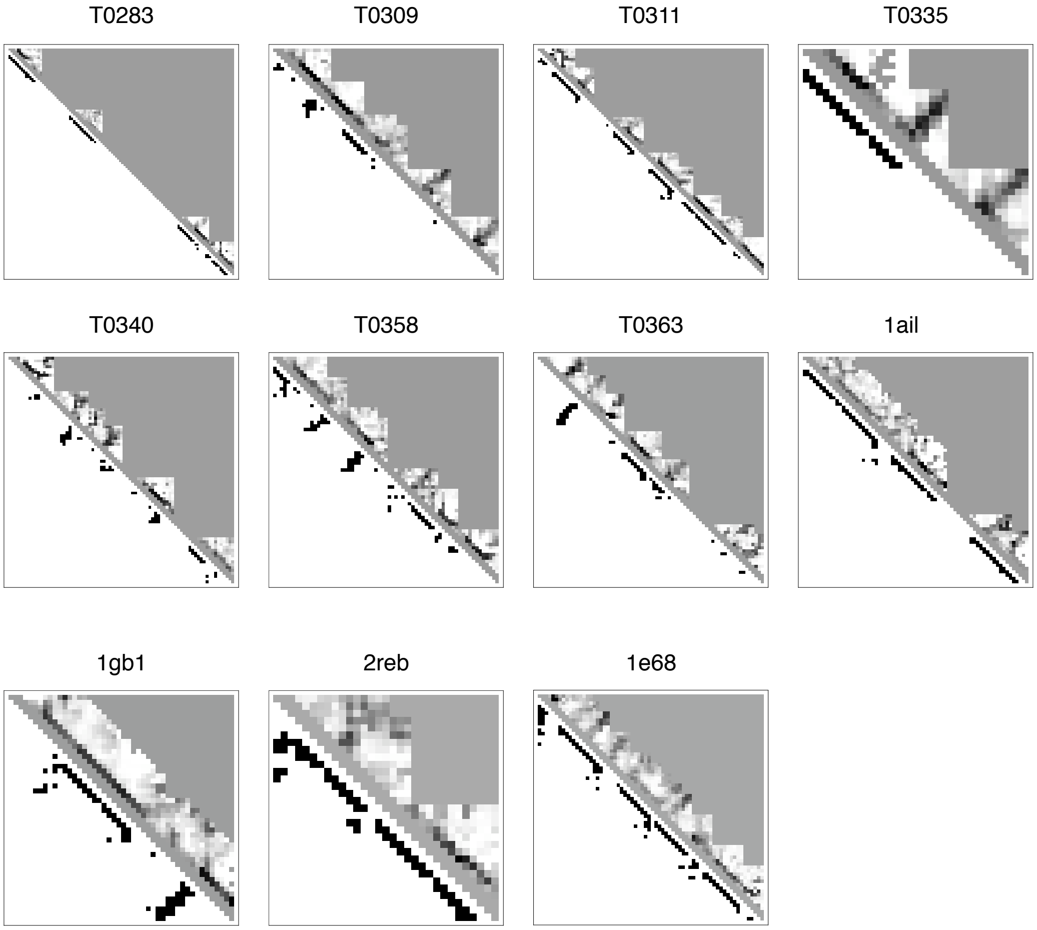

Fig. 17. Contact prediction 'logit' scores of the best regression models trained on 126mer simulations, for all proteins in our test set. Shading is as described in Figure 5 of the main text.

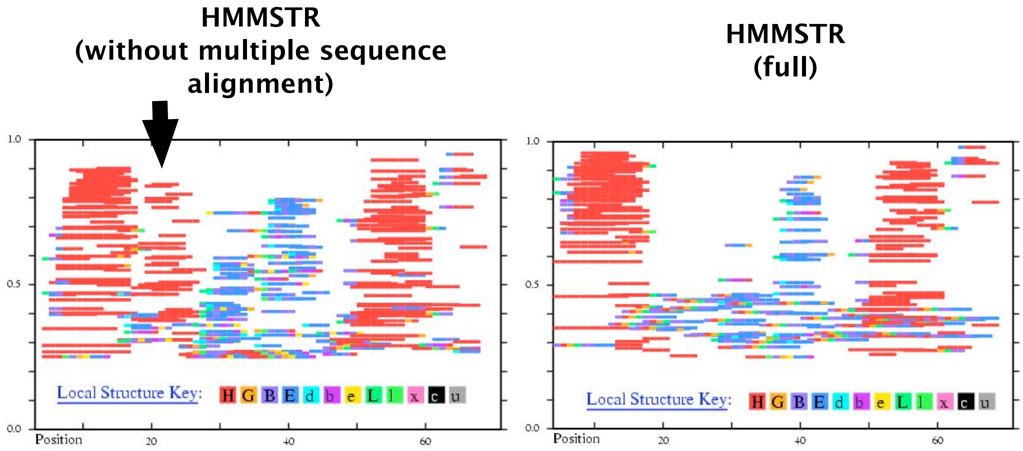

Fig. 18. **The I-sites library gives a high confidence value to a helical decoy structure we found in our ZAM fragment simulations.** *Left:* When multiple sequence alignments by PSI-BLAST are not allowed, the top I-sites motifs and top 5 HMMSTR/Robetta server predictions predict the same helical decoy structure (cyan) we find using molecular simulation. *Right:* When multiple sequence alignment is used in the prediction, the decoy helix gets filtered out.

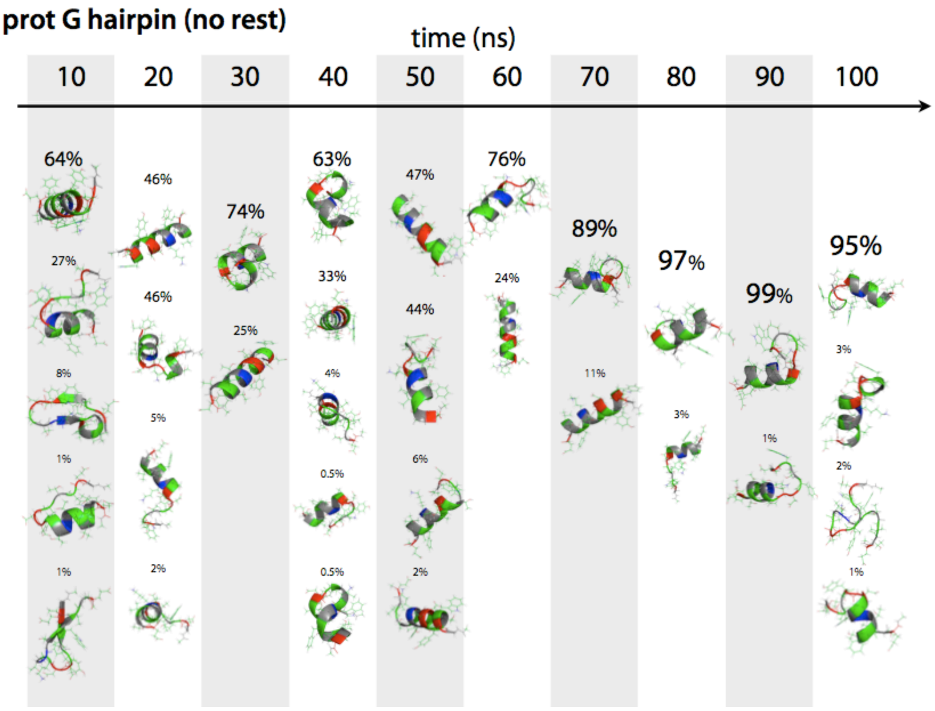

Fig. 19. **100 ns simulation of the C-terminal fragment of protein G (no restraints).**

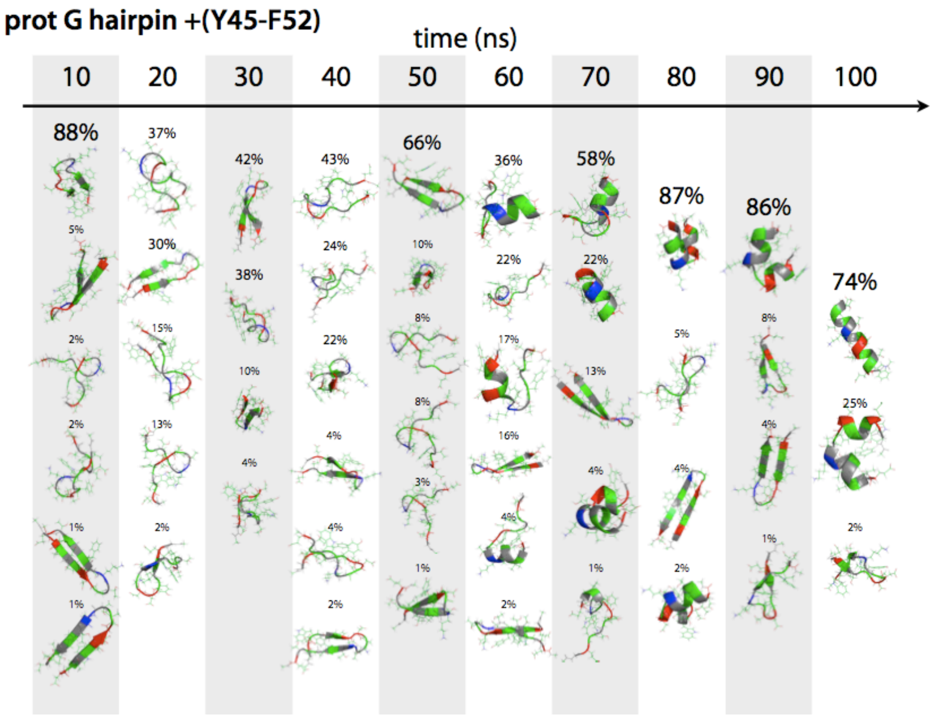

Fig. 20. **100 ns simulation of the C-terminal fragment of protein G, with (Y45,F52) restrained.**

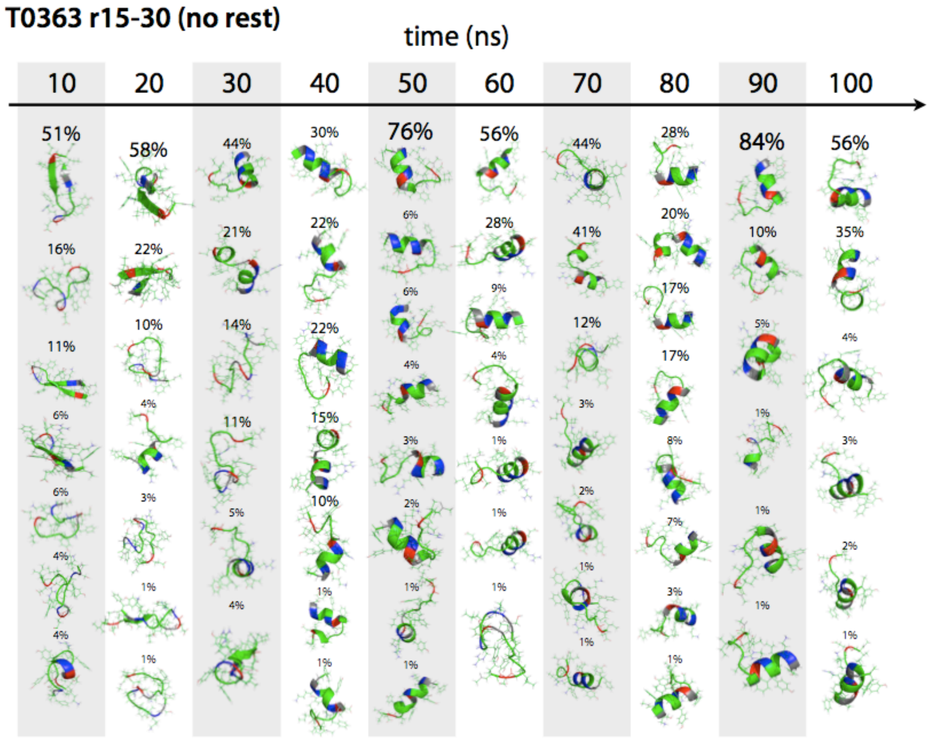

Fig. 21. **100 ns simulation of the T0363 fragment (no restraints).**

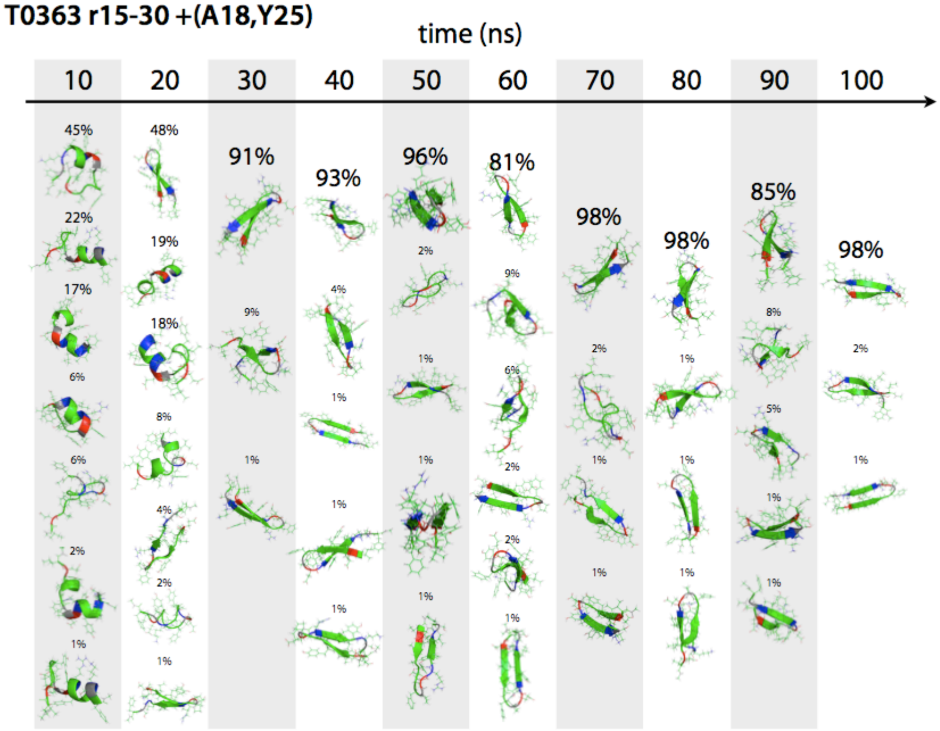

Fig. 22. **100 ns simulation of the T0363 fragment, with (A18,Y25) restrained**

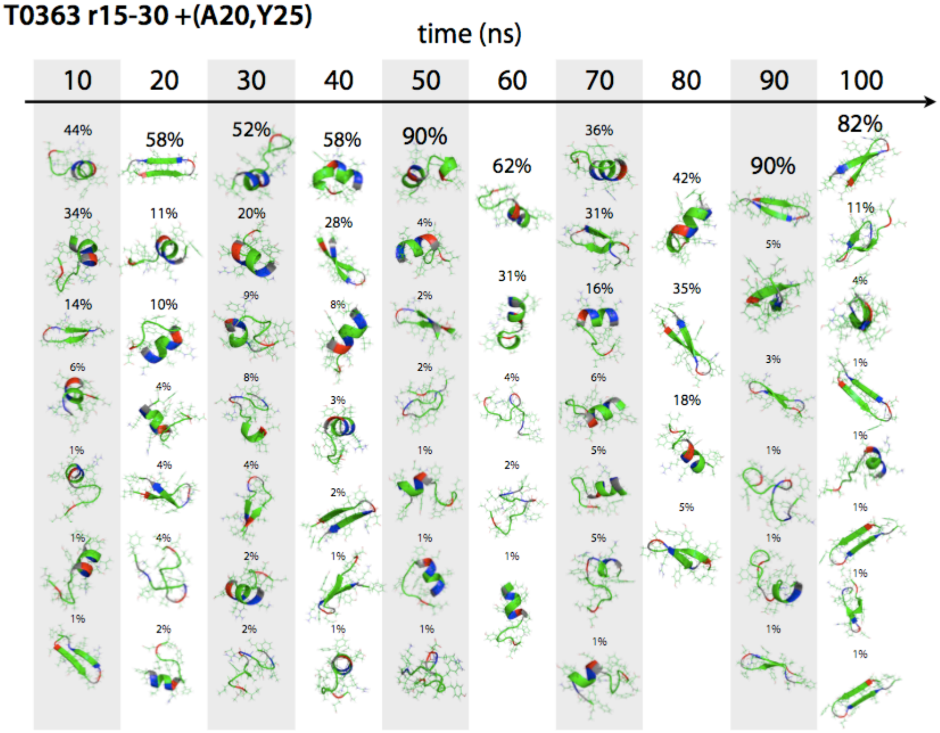

Fig. 23. **100 ns simulation of the T0363 fragment, with (A20,Y25) restrained**

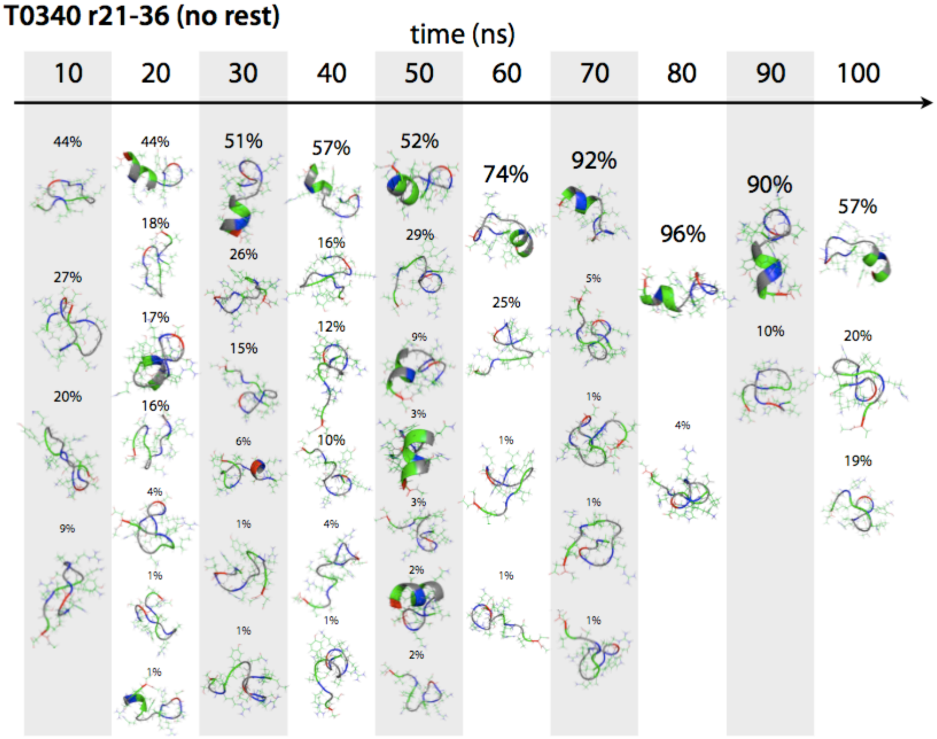

Fig. 24. **100 ns simulation of the T0340 fragment (no restraints).**

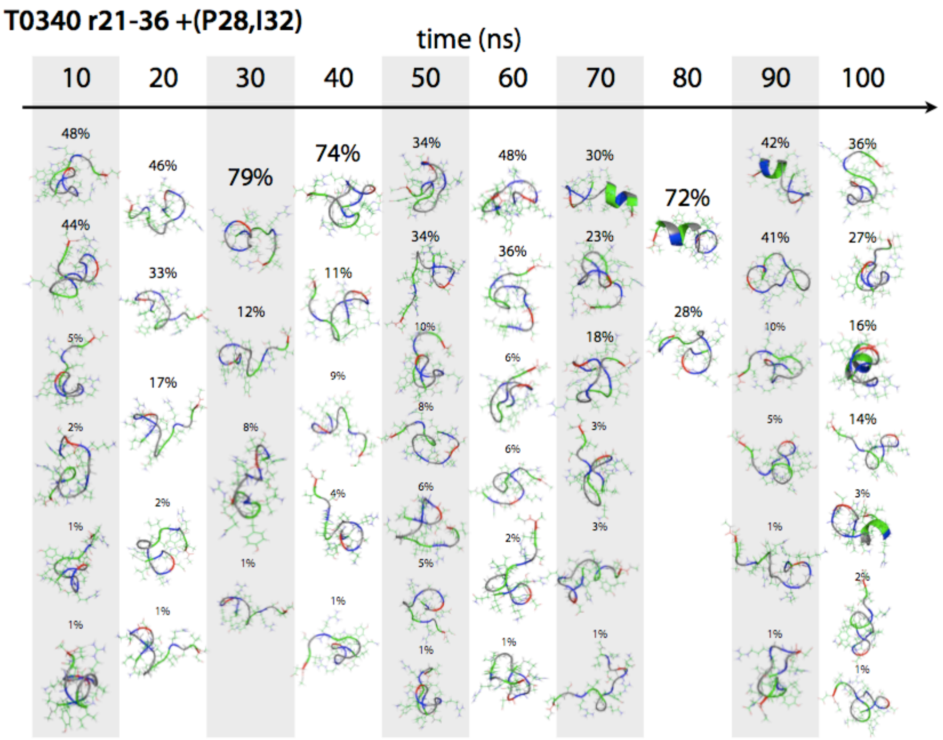

Fig. 25. **100 ns simulation of the T0340 fragment, with (P28,I32) restrained.**

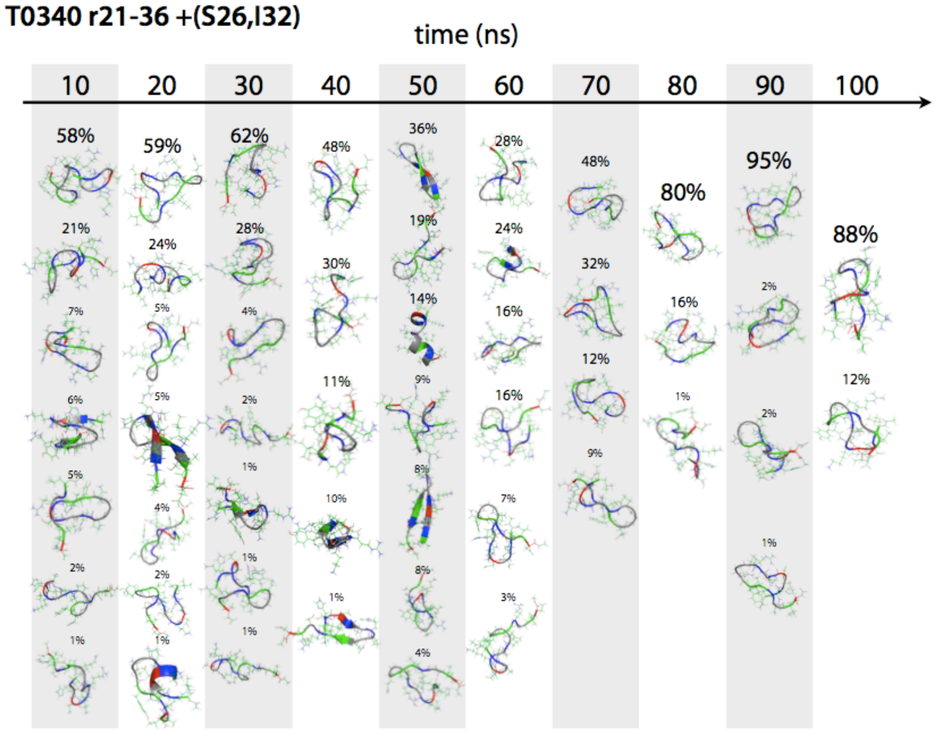

Fig. 26. **100 ns simulation of the T0340 fragment, with (S26,I32) restrained.**

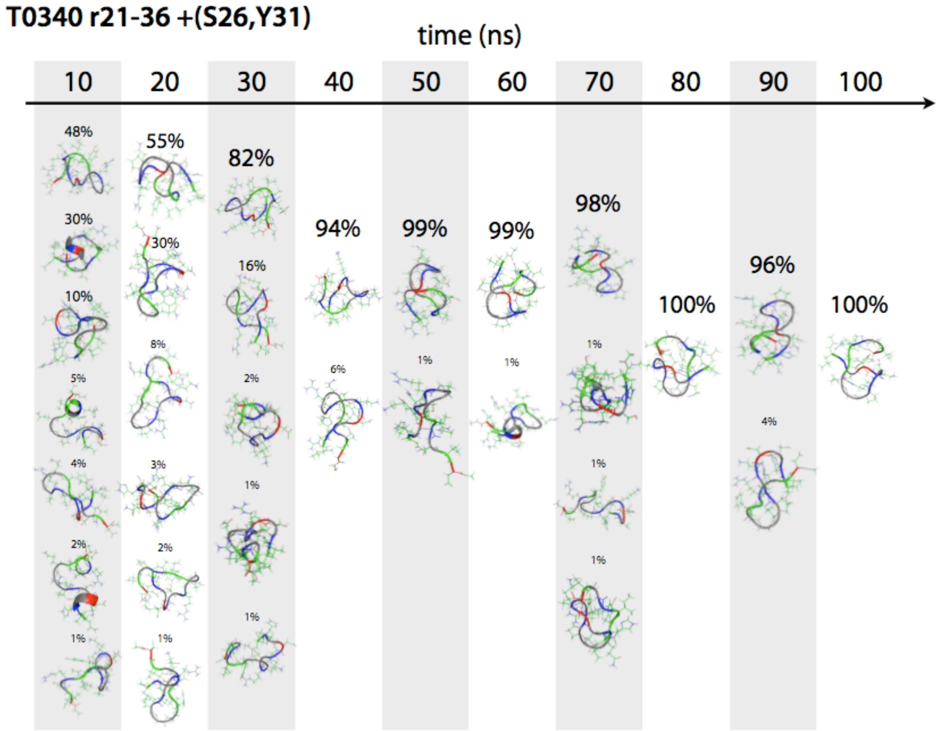

Fig. 27. **100 ns simulation of the T0340 fragment, with (S26,Y31) restrained.**

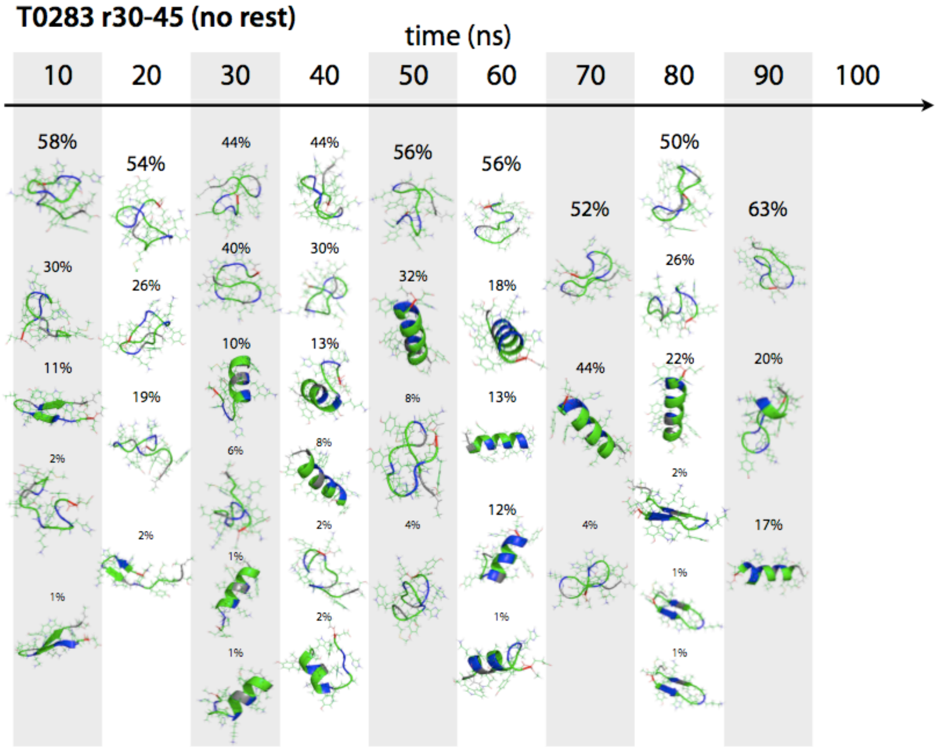

Fig. 28. **100 ns simulation of the T0283 fragment (no restraints).**

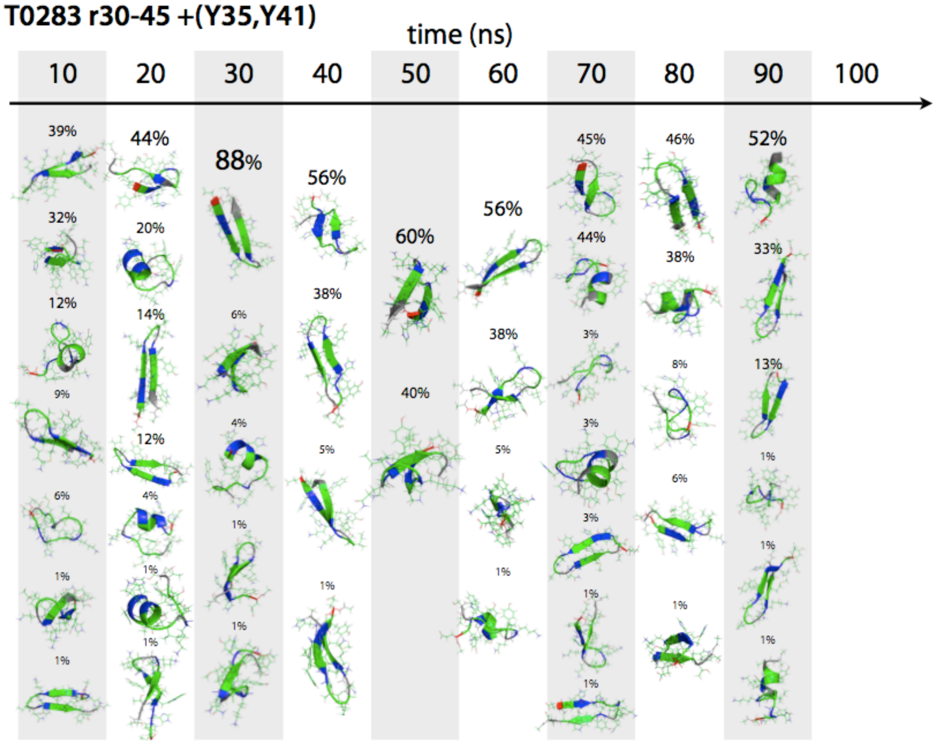

Fig. 29. **100 ns simulation of the T0283 fragment, with (Y35,Y41) restrained.**

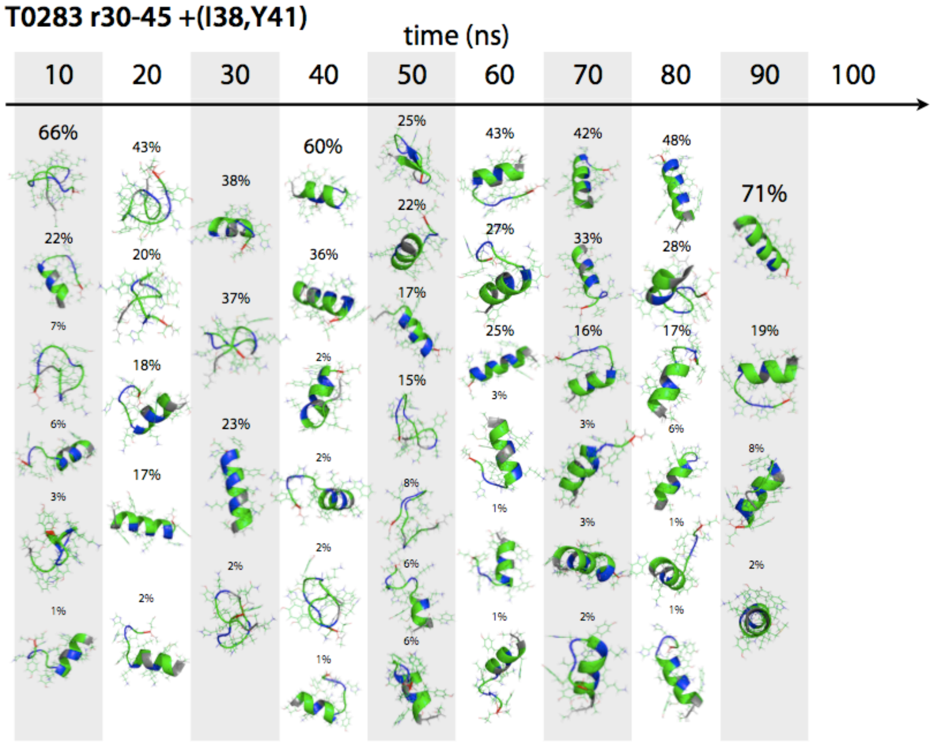

Fig. 30. **100 ns simulation of the T0283 fragment, with (I38,Y41) restrained.**

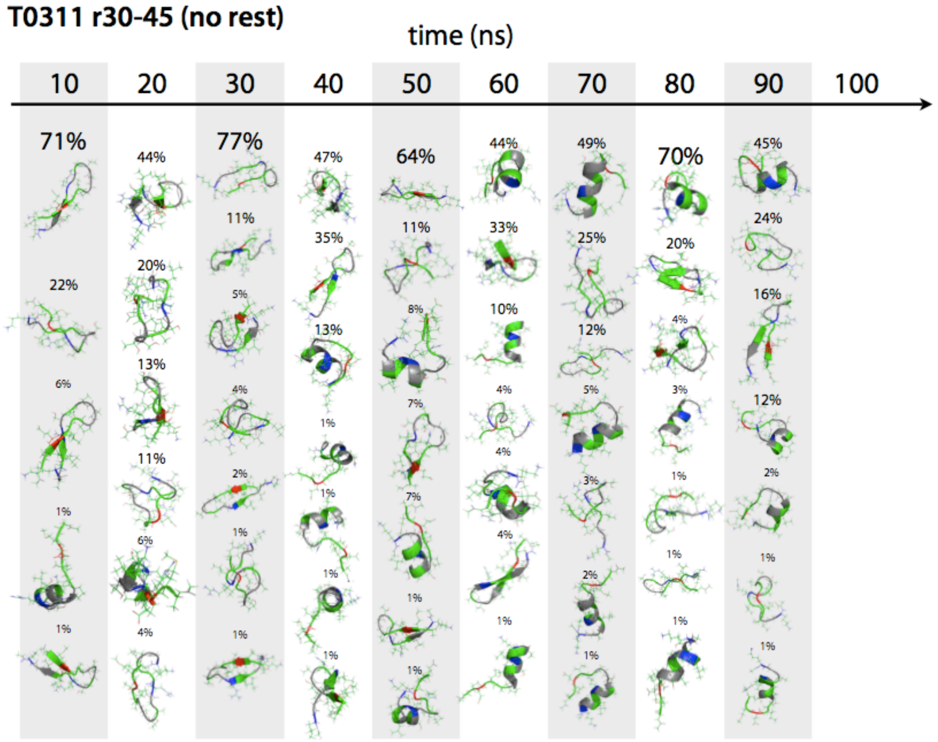

Fig. 31. **100 ns simulation of the T0311 fragment (no restraints).**

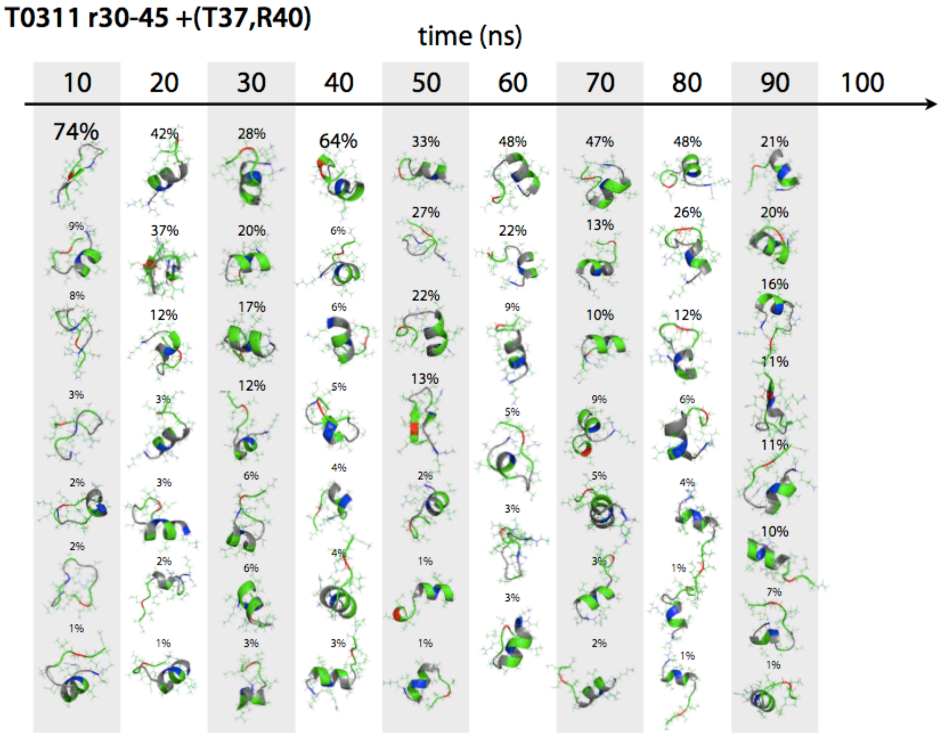

Fig. 32. **100 ns simulation of the T0311 fragment, with (T37,R40) restrained.**

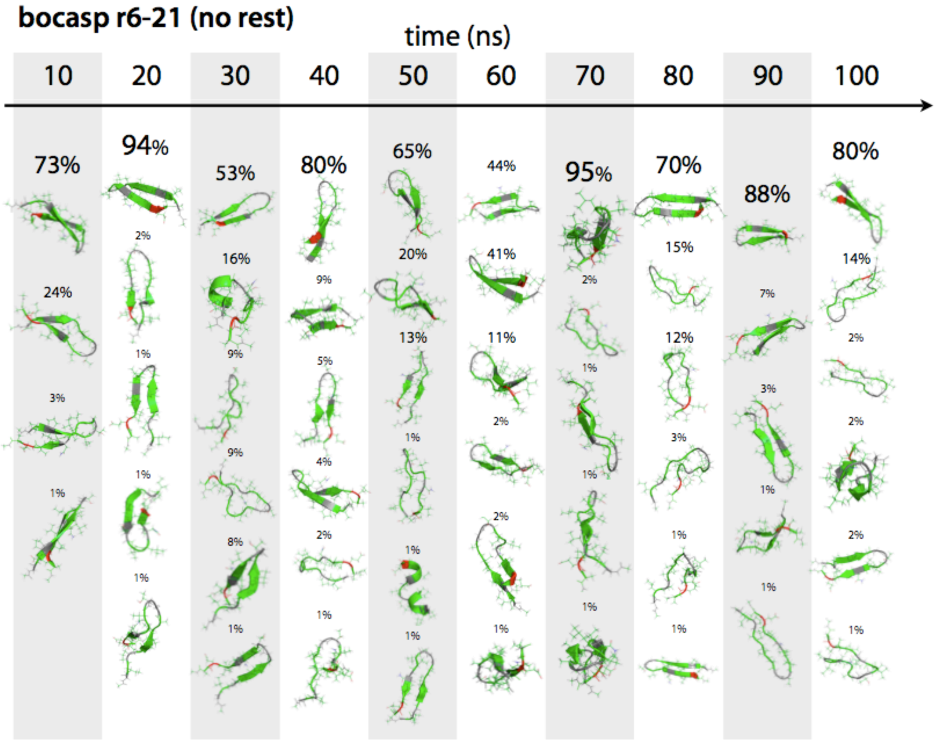

Fig. 33. **100 ns simulation of the 1e68 protein fragment, residues 6-21 (no restraints).**

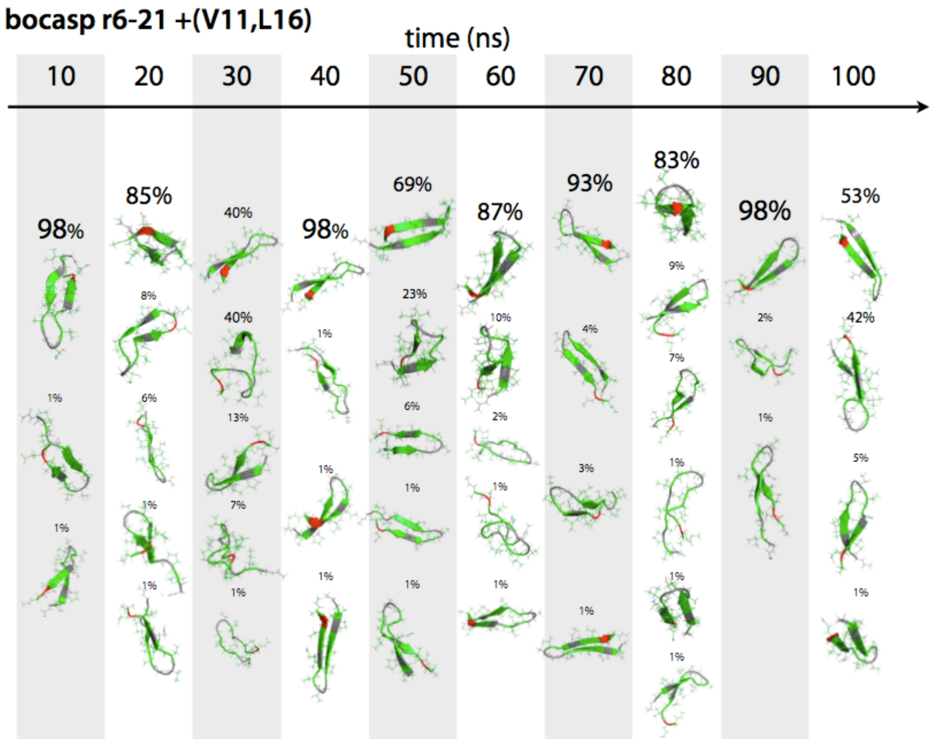

Fig. 34. **100 ns simulation of the 1e68 protein fragment, residues 6-21, with (V11,L16) restrained.**

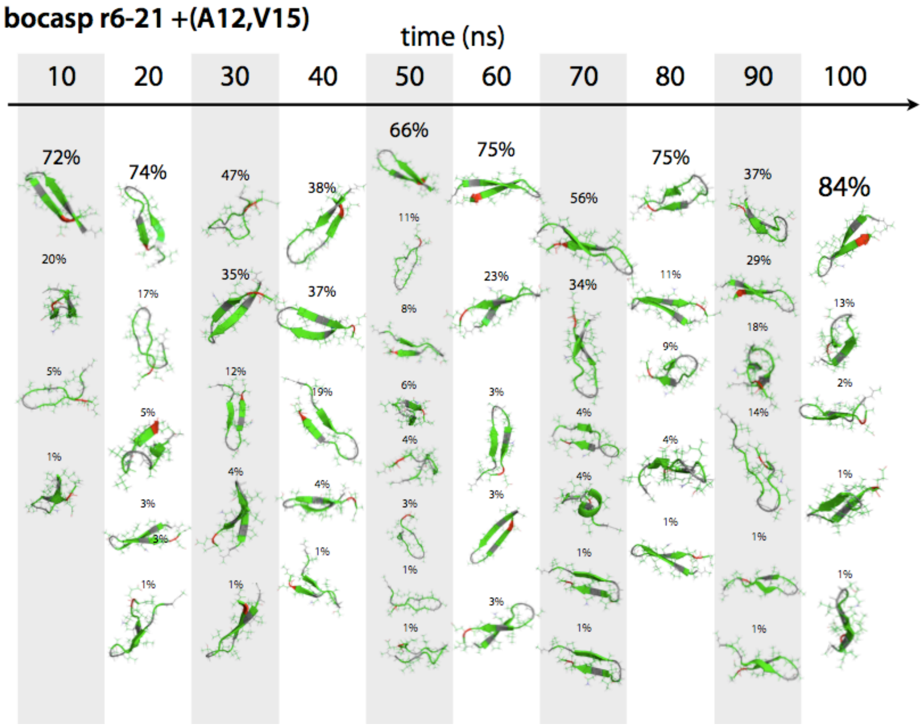

Fig. 35. **100 ns simulation of the 1e68 protein fragment, residues 6-21, with (A12,V15) restrained.**

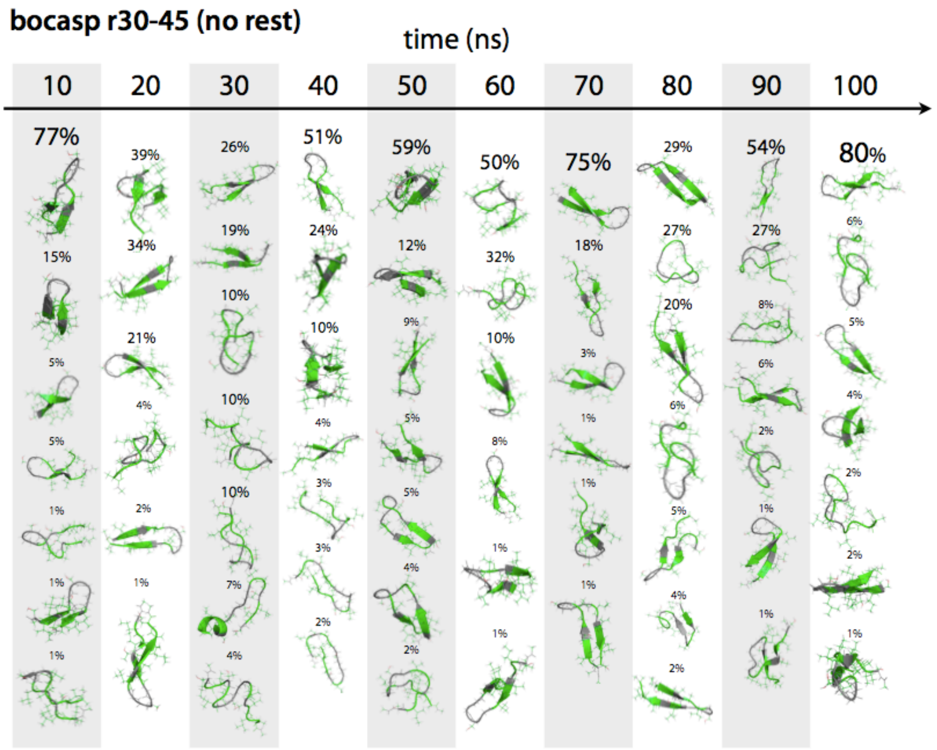

Fig. 36. **100 ns simulation of the 1e68 protein fragment, residues 30-45 (no restraints).**

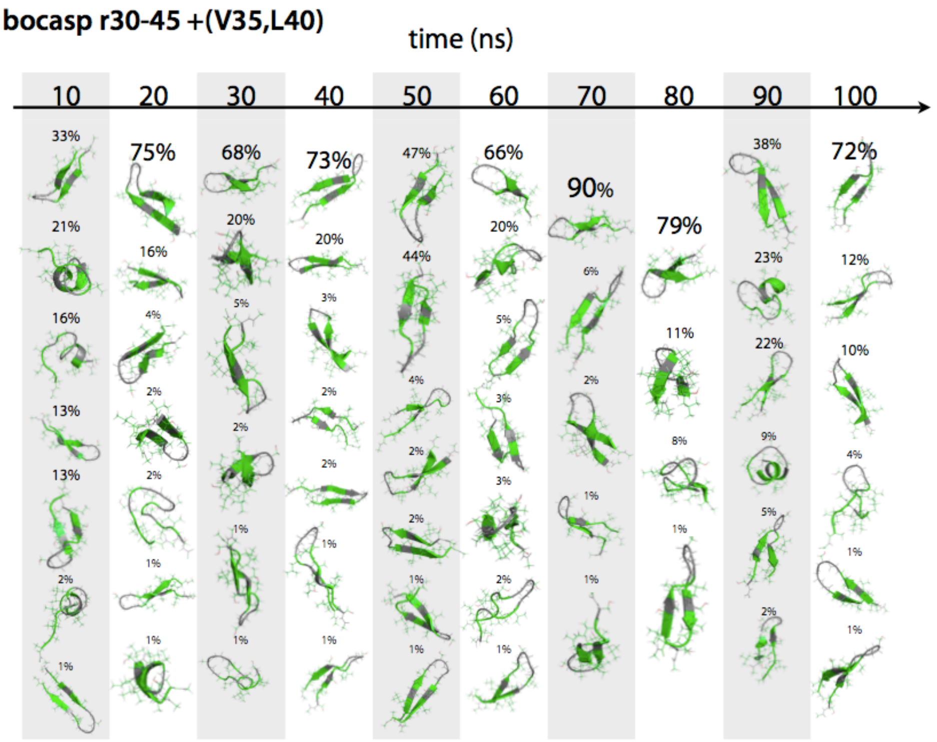

Fig. 37. **100 ns simulation of the 1e68 proteinfragment, residues 30-45, with (V35,L40) restrained.**

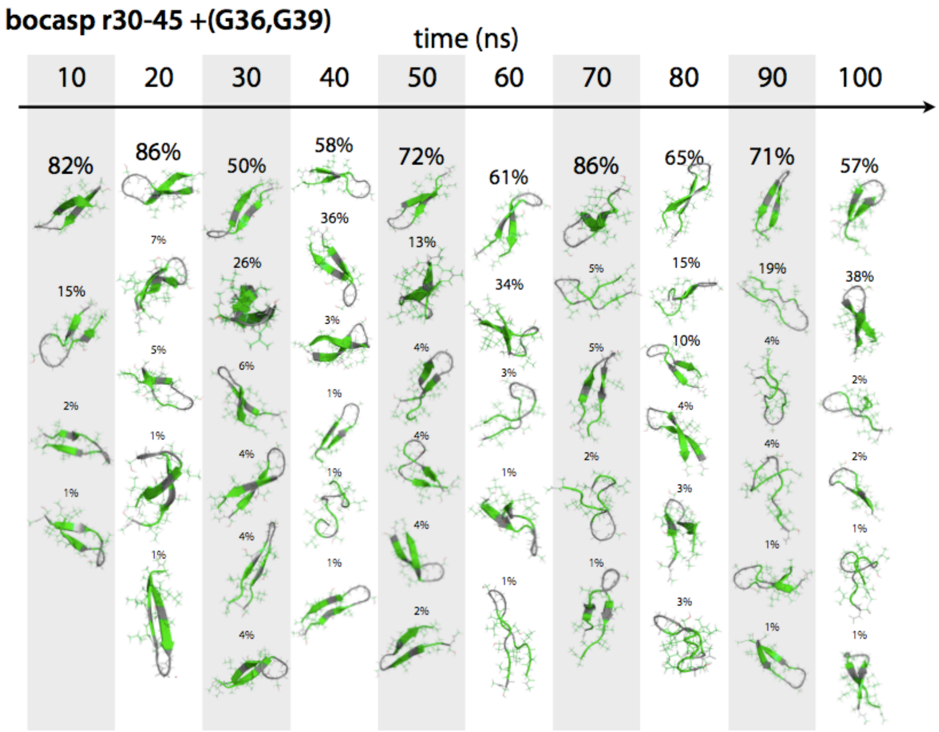

Fig. 38. **100 ns simulation of the 1e68 protein fragment, residues 30-45, with (G36,G39) restrained.**
